# Matrix Architecture and Integrin Branch Balance Distinguish Immune-Regulatory States in Clear Cell Renal Cell Carcinoma

**DOI:** 10.3390/cimb48080789

**Published:** 2026-08-02

**Authors:** Caner Karaca, Mehmet Emin Arayici, Hüseyin Salih Semiz, Hulya Ellidokuz, Yasemin Basbinar

**Affiliations:** 1 Department of Translational Oncology, Institute of Oncology, Dokuz Eylul University, 35330 Izmir, Türkiye; 2 Department of Biostatistics and Medical Informatics, Faculty of Medicine, Dokuz Eylul University, 35330 Izmir, Türkiye; 3 Department of Preventive Oncology, Institute of Oncology, Dokuz Eylul University, 35330 Izmir, Türkiye

**Keywords:** ccRCC, matrix reorganization, integrin mechanosensing, RhoA/ROCK signaling, SRC signaling, adenosine-mediated immune resistance, CD276, tumor microenvironment

## Abstract

Background/Objectives: Clear cell renal cell carcinoma (ccRCC) is frequently vascular and immune-infiltrated, yet durable responses to immune checkpoint blockade remain limited. This suggests that immune resistance may reflect tumor microenvironmental organization and mechanotransduction state rather than immune infiltration alone. We aimed to determine whether matrix reorganization and branch-specific integrin mechanosensing define immune-regulatory states in ccRCC, with particular attention to adenosine-associated immune resistance. Methods: We performed an integrative computational analysis of TCGA-KIRC bulk RNA-sequencing, clinical, survival, immune feature, and reverse-phase protein array data. Matrix- and mechanobiology-related programs were quantified using ssGSEA, compact z-score-based signatures, and principal component-based sensitivity analyses. Immune-regulatory programs, CAF and ECM scores, FAK/SRC activation features, and MINER-inferred transcriptional regulons were integrated using stage association, correlation, partial correlation, variance partitioning, survival, and transcriptional state analyses. Results: Matrix-centered transcriptional programs were the dominant stage-associated mechanobiology signal in ccRCC, including ECM deposition, collagen organization, matrix remodeling, fluid shear stress, and YAP/TAZ activity. A compact ECM-associated core (ECM_Stiffness_Core; a ten-gene signature whose highest-loading members include FN1, COL1A1, COL6A1, and LOX) captured a matrix reorganization program, indicating remodeling of ECM composition and architecture rather than uniform increases in tumor stiffness, pressure, or bulk mechanical load. Matrix remodeling was associated with CAF abundance, TGFβ signaling, CD276/B7-H3, CSF1-related myeloid biology, ENTPD1/CD39, and PRDM1, whereas associations with cytotoxic immune cells were weaker. Integrin mechanosensing separated into opposing branches: ITGA5/ILK/SRC-associated features aligned with higher-risk biology and adenosine-linked immune regulation, whereas PTK2/FAK–RHOA–ROCK components showed lower-risk directions. RPPA analyses supported SRC–FAK imbalance as an adverse signaling pattern. MINER analyses further separated matrix-associated immune-suppressive regulons from canonical integrin/focal adhesion states. Conclusions: Matrix reorganization and integrin branch imbalance appear to shift ccRCC toward distinct immune-regulatory states. We propose a conceptual model that matrix architecture may act as a directional suppressive amplifier, whereas the relative balance between ITGA5/ILK/SRC-associated signaling and canonical PTK2/FAK–RHOA–ROCK mechanosensing functions as an integrin branch rheostat. This framework identifies matrix remodeling, CD276/B7-H3, CSF1-related myeloid biology, adenosine signaling, and SRC–FAK imbalance as candidate biological axes for future investigation, including their potential relevance to combination strategies beyond PD-1/PD-L1 blockade. Future experimental, spatial, and treatment response studies may further clarify the mechanistic basis of these associations and evaluate their potential therapeutic relevance.

## 1. Introduction

Clear cell renal cell carcinoma (ccRCC) is the most common histological subtype of renal cell carcinoma and is characterized by recurrent disruption of oxygen-sensing, angiogenic, metabolic, chromatin-remodeling, and immune-regulatory pathways [1]. The treatment landscape of advanced ccRCC has been transformed by immune checkpoint inhibitor-based combinations and VEGF/VEGFR-targeted therapies, reflecting the central importance of both antitumor immunity and angiogenesis in this disease [2,3,4,5]. Nevertheless, a substantial proportion of patients fail to achieve durable benefit from immune checkpoint blockade, indicating that ccRCC resistance is not explained solely by the presence or absence of PD-1/PD-L1 signaling [6].

Resistance to immunotherapy is increasingly understood in terms of spatial and functional immune phenotypes, including immune-inflamed, immune-excluded, and immune-desert tumor microenvironments [7,8]. In immune-desert tumors, effector immune cells are scarce within the tumor bed, whereas immune-excluded tumors may contain immune cells that remain spatially restricted to stromal or peritumoral compartments rather than productively entering tumor cell regions [8]. This distinction is particularly relevant in ccRCC, which is often immune-infiltrated but not necessarily immune-effective, suggesting that resistance may arise from the organization, localization, and functional state of immune cells rather than from simple absence of immune infiltration [6]. Mechanisms that reshape the stromal architecture of ccRCC may be central determinants of immune exclusion and incomplete response to checkpoint inhibition.

The molecular biology of ccRCC is strongly linked to loss of the von Hippel–Lindau tumor suppressor gene, which disrupts oxygen-dependent degradation of hypoxia-inducible factor alpha subunits [9,10]. VHL loss stabilizes HIF signaling and establishes a pseudohypoxic transcriptional state that promotes angiogenesis, metabolic rewiring, tumor adaptation, and VEGF-driven vascularization [11,12]. This biology explains the highly vascular nature of ccRCC and provides therapeutic rationale for the inhibition of the VEGF pathway, but it does not fully account for why vascular, immune-infiltrated tumors can remain clinically resistant to immune checkpoint blockade [4,6]. Additional tumor microenvironmental features are needed to explain the coexistence of angiogenesis, immune infiltration, immune dysfunction, and treatment resistance in ccRCC.

Extracellular matrix remodeling is one such candidate mechanism. Across solid tumors, ECM deposition, collagen crosslinking, and matrix stiffening can promote malignant progression by increasing integrin signaling, focal adhesion activation, cytoskeletal tension, invasion, and resistance to therapy [13,14]. In ccRCC, intratumoral fibrosis is a frequent histopathological feature and is associated with adverse clinicopathological factors, including higher nuclear grade, lymphovascular invasion, and intratumoral necrosis [15]. A broader review of renal cell carcinoma fibrosis further supports the clinical relevance of fibrotic signaling, including TGFβ, EMT, CAF activation, mTOR, WNT, Hippo/YAP, collagen remodeling, LOX-family activity, and matrix-associated therapeutic resistance [16]. Together, these studies suggest that matrix remodeling is not merely a passive histological consequence of tumor growth, but a biologically active component of ccRCC progression.

Several observations make collagen and LOX-family remodeling especially relevant to ccRCC. Joung et al. reported intratumoral fibrosis in most of the examined ccRCC specimens and detected LOX expression in the tumor cell cytoplasm in more than half of the cases, supporting a direct link between ccRCC tissue architecture and matrix crosslinking biology [15]. Fibrotic signaling reviews of RCC describe CAFs as important producers of collagen, fibronectin, tenascin C, periostin, MMPs, and LOX-family enzymes, which collectively remodel the ECM and may promote invasion, drug resistance, angiogenesis, and immune suppression [16]. COL1A1, fibronectin, collagen VI, and LOX therefore represent not only structural matrix markers, but also components of a broader matrix organization program that can connect stromal activation to tumor cell mechanosensing and immune-regulatory signaling [13,16].

However, matrix biology in ccRCC should not be interpreted through a simple model in which aggressive tumors are uniformly stiff. Unlike classical desmoplastic tumors such as pancreatic or breast cancer, ccRCC is highly vascular, lipid-rich, spatially heterogeneous, and often mechanically complex. Prior mechanical studies suggest that renal tumor stiffness may vary by measurement context and that RCC tissue is not necessarily uniformly stiffer than adjacent non-tumor kidney [17,18]. Thus, the key biological feature in ccRCC may be local matrix reorganization rather than a global increase in the bulk elastic modulus. Collagen alignment, fibronectin-rich scaffolding, LOX-mediated crosslinking, and CAF-associated matrix remodeling may create focal mechanosensitive niches even when the tumor as a whole does not behave as a uniformly stiff mass [13,16].

Matrix remodeling may also help explain immune exclusion. In multiple tumor contexts, dense or reorganized ECM can restrict immune cell migration, alter cytokine and growth factor gradients, activate CAF programs, and promote myeloid- and checkpoint-associated immune suppression [14]. TGFβ-rich stromal programs have been shown to support T-cell exclusion and resistance to checkpoint blockade in other solid tumors, providing a conceptual framework for matrix-associated immune resistance [19,20]. In ccRCC specifically, intratumoral fibrosis has been linked to immune infiltration patterns and checkpoint-related immune features, while reviews of fibrotic signaling highlight the overlap among CAF activation, TGFβ signaling, ECM remodeling, angiogenesis, and immune regulation [15,16]. These findings support the hypothesis that fibrotic ccRCC may not be immunologically “cold,” but may instead represent an immune-excluded or immune-dysfunctional state in which immune cells are present yet spatially and functionally constrained.

Beyond the canonical PD-1/PD-L1 axis, alternative immune-suppressive pathways may be particularly important in matrix-remodeled tumors. B7-H3, encoded by CD276, is a B7-family immune-regulatory molecule that has emerged as a therapeutic target across solid tumors and may contribute to immune suppression, tumor invasion, angiogenesis, and resistance phenotypes [21,22]. Adenosine signaling, generated by ectonucleotidases such as CD39/ENTPD1 and CD73/NT5E and transmitted via adenosine receptors, represents another hypoxia- and stress-linked immune checkpoint that can suppress effector T-cell and NK-cell function within the tumor microenvironment [23].

Mechanistically, ECM cues are transmitted through integrins, focal adhesion complexes, SRC-family kinases, FAK/PTK2, ILK, Rho-family GTPases, actomyosin contractility, and YAP/TAZ–TEAD transcriptional programs [24]. Yet these downstream branches do not necessarily carry identical biological meaning. A fibronectin–integrin–SRC axis may promote adhesion-adaptive tumor cell survival, invasion, and plasticity, whereas canonical FAK/RhoA/ROCK signaling may behave differently depending on tissue context, cell state, and stromal architecture. Therefore, ccRCC mechanotransduction should be examined as branch-specific signaling rather than as a uniform “mechanosensing-high” state. This is especially relevant for distinguishing stromal matrix production from tumor cell-intrinsic mechanotransduction.

On this basis, we hypothesized that intratumoral matrix reorganization in ccRCC defines a stromal immune-remodeled phenotype that is not explained by immune infiltration alone. We further proposed that this phenotype would be organized by separable but interacting mechanobiology axes: a matrix architecture program characterized by collagen-, fibronectin-, and LOX-associated ECM remodeling, and an integrin-linked mechanosensing program in which distinct downstream branches may carry different biological and immune-regulatory meanings. By integrating transcriptomic, immune-regulatory, RPPA-based signaling, survival, and MINER regulon-state analyses in TCGA-KIRC, this study tests whether matrix remodeling and integrin branch balance explain immune-suppressive remodeling in ccRCC, with particular attention paid to CD276/B7-H3, CSF1-associated myeloid biology, TGFβ/stromal regulation, adenosine signaling, and SRC–FAK-associated focal adhesion rheostat as candidate co-targetable resistance axes.

## 2. Materials and Methods

### 2.1. Study Design and Data Sources

This study performed an integrative computational analysis of the TCGA Kidney Renal Clear Cell Carcinoma (TCGA-KIRC) cohort to relate mechanobiology-associated transcriptional programs to immune-regulatory phenotypes, transcriptional regulatory networks, and clinical outcomes. Bulk RNA-sequencing, clinical, survival, and reverse-phase protein array (RPPA) data were integrated at the TCGA sample or participant barcode level. All analyses used publicly available de-identified data and were performed in R (with the modern GSVA 2.0.7 API; package builds under R 4.4.3) and Python (3.12.2).

### 2.2. RNA-Sequencing Data Acquisition and Preprocessing

STAR–Counts-derived gene-level unstranded counts and TPM matrices for TCGA-KIRC were obtained from the UCSC Xena GDC hub. Ensembl gene identifiers were stripped of version suffixes and annotated against GDC rowRanges biotype metadata; genes were classified by gene_biotype and analyses were restricted to protein_coding genes. Duplicate Ensembl identifiers were resolved with distinct (ensembl_gene_id, keep_all = TRUE), identifiers were collapsed to HGNC symbols, and symbols were upper-cased. Primary tumor and adjacent-normal samples were separated by TCGA sample-type code. TPM values were transformed as log2(TPM + 1). Before z-scoring and downstream enrichment/correlation/network analyses, genes producing undefined z-scores (zero variance) were removed via a t(scale(t(.))) check, yielding a clean protein-coding matrix of 18,393 genes × 535 tumor samples. Per-gene z-scores across tumor samples were computed as t(scale(t(matrix))) and reused throughout as gene_z.

### 2.3. Clinical, Survival, and Sample Annotation

Clinical annotations came from the UCSC Xena GDC TCGA-KIRC clinical table; AJCC pathologic stage was harmonized into Stage I–IV by string matching (str_detect, longest-pattern-first to avoid “Stage I”/“Stage II” collisions), and tumor grade and tissue type were retained. Overall survival (OS) and progression-free interval (PFI) endpoints were taken from UCSC Xena survival annotations and the Thorsson et al. PanImmune resource and merged by participant barcode. RNA-seq, clinical, survival, RPPA, and immune feature tables were matched on harmonized TCGA barcodes (periods converted to hyphens).

Mechanobiology gene sets and signature scoring. Two complementary scoring strategies were used. (i) Curated MSigDB pressure/mechanotransduction gene sets were retrieved with msigdbr (species Homo sapiens), upper-cased, and scored by single-sample gene set enrichment analysis using GSVA ssgseaParam(exprData = pcoding_tpm_matrix, geneSets = …, minSize = 9, maxSize = 500, alpha = 0.25, normalize = TRUE), executed via gsva() (GSVA v2.0.7; 27 gene sets retained after size filtering). Enrichment scores were z-scored per signature across samples (t(scale(t(.)))), and aggregate module scores were the mean of constituent signature z-scores. (ii) To define compact, interpretable axes, custom signatures were scored as the per-sample mean of gene-wise z-scores using gene_z. The custom signatures were: ECM_Stiffness_Core (FN1, COL1A1, COL1A2, COL6A1, COL6A2, COL6A3, LOX, LOXL2, POSTN, TNC), Integrin_Mechanosensing (ITGB1, ITGA5, ITGAV, PTK2, SRC, ILK, RHOA, ROCK1, ROCK2), YAP_TAZ_Mechanosensing (YAP1, WWTR1, CCN2, CCN1, ANKRD1, AMOTL2), Interstitial_Fluid_Pressure (AQP1, VEGFA, PDPN, LYVE1, PROX1), and Matrix_Remodeling (MMP2, MMP9, MMP14, TIMP1, TIMP2, TGFB1, ACTA2). For the branch balance analysis, Integrin_Mechanosensing was decomposed into two functionally defined sub-branches, scored by the same mean gene-wise z-score method: an upstream ITGA5/ILK/SRC branch (ITGA5, ILK, SRC) and a downstream PTK2/FAK–RHOA–ROCK branch (PTK2, RHOA, ROCK1, ROCK2). As a sensitivity analysis, each signature was also scored by its first principal component (prcomp, center = TRUE, scale = TRUE); PC1 sign was oriented to correlate positively with the corresponding mean z-score via Spearman correlation (flipped when ρ < 0), and a composite mechanosensing index (CMI) was derived from per-axis PC1 scores.

### 2.4. Gene Set Resources and Mechanobiology Signature Scoring

Matrix- and mechanobiology-related gene sets were assembled from curated pathway resources and custom axis-specific signatures. These included integrin signaling, FAK/RhoA signaling, ECM and collagen organization, matrix remodeling, EMT, YAP/TAZ activity, and fluid shear stress programs.

Single-sample gene set enrichment analysis was performed using the GSVA package with the ssGSEA method. Enrichment scores were z-scored across samples for visualization and downstream comparisons. Aggregate module scores were calculated as the mean of constituent signature z-scores.

To define more compact and interpretable mechanobiology axes, axis-specific signatures were additionally scored as the per-sample mean of gene-wise z-scores, using log2(TPM + 1) expression values z-scored across tumor samples. ssGSEA (GSVA) was applied only to the MSigDB pathway sets (Supplementary Figure S1) and not to the custom axis signatures. For the descriptive comparison of adjacent-normal kidney versus tumor stages, gene-wise z-scores were computed across the combined normal-plus-tumor sample set so that both tissue types share a common scale. For all tumor-only analyses, gene-wise z-scores were computed across tumor samples only. Principal component-based scoring was performed as a sensitivity analysis. For each signature, the first principal component was calculated from centered and scaled expression values, and the sign of PC1 was oriented to correlate positively with the corresponding mean z-score. A composite mechanosensing index was derived from the PC1 scores of the individual mechanobiology axes.

### 2.5. RPPA Protein Expression Processing and Mechanoactivation Scores

Level-4 log2-normalized RPPA protein-by-sample data for TCGA-KIRC were obtained from UCSC Xena (487 antibodies × 478 samples, comprising adjacent-normal and Stage I–IV tumor samples) and harmonized to the RNA-seq barcode convention. These are TCPA/MD Anderson Level-4 replicate-base-normalized (RBN) values, in which each antibody is independently quantified by SuperCurve fitting and then median-centered twice (per protein across samples, then per sample across proteins), placing all antibodies on a common, dimensionless log2 scale. Because every antibody is therefore already normalized on the same scale, phospho-to-total and active-to-inactive ratios were computed as simple differences between the two normalized log2 values (e.g., FAK_pY397 − FAK), corresponding to log2 fold-differences; no additional per-antibody standardization was applied before ratio computation. Tier-1 ratios were FAK_activation (FAK_pY397 − FAK), SRC_activation (SRC_pY416 − SRC_pY527), YAP_activity (YAP − YAP_pS127), Myosin_contractility (MYOSINIIA_pS1943 − MYOSINIIA), AKT_activation (AKT_pS473 − AKT), and mTOR_activation (MTOR_pS2448 − MTOR); additional ratios included GSK3_inhibition (GSK3ALPHABETA_pS21S9 − GSK3ALPHABETA) and canonical and non-canonical EphA2 (EphA2_pY588 − EphA2; EphA2_pS897 − EphA2). A Composite Mechanoactivation Score (CMAS) was defined as the first principal component of the six Tier-1 ratios (*n* = 260 samples with complete Tier-1 coverage; PC1 variance explained = 31.2%), oriented to correlate positively with FAK_activation. Derived ratio scores were standardized to unit standard deviation before entering Cox models. Of the profiled samples, 475 were staged tumors; FAK and SRC activation ratios were both computable for 255 tumors, of which 254 with complete survival data were used for the FAK/SRC survival and quadrant analyses. FAK/SRC quadrants were defined by an independent median split on FAK_activation and SRC_activation, yielding FAK-low/SRC-low (n = 67), FAK-high/SRC-low (n = 60), FAK-low/SRC-high (n = 60), and FAK-high/SRC-high (n = 67). RNA–RPPA concordance analyses used the intersection of tumor samples profiled on both platforms (n = 471).

### 2.6. Immune Landscape Characterization

Immune features were assembled from three sources. Published TCGA-KIRC immune metrics (immune subtype, leukocyte fraction, lymphocyte infiltration, IFN-γ and TGF-β response signatures, CIBERSORT cell fractions) were taken from Thorsson et al. Stromal/immune abundance was estimated by GSVA with MCP-counter and xCell marker sets on the tumor matrix. Curated immune-regulatory panels were scored directly from log2(TPM + 1)/gene_z, including inhibitory checkpoints, co-stimulatory genes, the adenosine pathway (ENTPD1/CD39, NT5E/CD73, ADORA2A, ADORA2B), Treg-associated genes (e.g., FOXP3, TNFRSF18, IL10, TGFB1), and exhaustion-associated transcription factors (TOX, TOX2, NR4A1/2, PRDM1, BATF, EOMES, TBX21). Composite scores included cytolytic activity (CYT = mean of GZMA, PRF1), the 18-gene T-cell-inflamed GEP (Ayers et al., including CD274 and CD276), and the CD8A/FOXP3 balance, plus total immune suppression and adenosine-activity scores.

### 2.7. Transcriptional Regulatory Network Inference with MINER

Transcriptional regulatory architecture was reconstructed with the MINER3 implementation (Mechanistic Inference of Node-Edge Relationships, Baliga Lab) applied to the protein-coding expression matrix [25]. MINER3 first identified and refined co-expression clusters and then linked these clusters to candidate transcription factor regulators through enrichment of shared binding-site evidence. A regulon was defined as a set of coherently expressed genes putatively controlled by a common regulator, thereby representing a candidate functional unit of transcriptional regulation. For each regulon, activity across samples was represented by its eigengene, defined as the first principal component of the expression profiles of its member genes.

Regulons showing similar activity patterns across tumors were grouped into transcriptional programs, representing coordinated higher-order regulatory processes. Tumors sharing similar global patterns of activated and repressed regulons and programs were assigned to transcriptional states, reflecting distinct network-level sample phenotypes. Bicluster membership was computed against a background model and transcriptional states were inferred from regulon activity patterns and programs assembled from co-expressed regulons. A bridge/relay backbone network was extracted by retaining bridge nodes with degree ≥ 50 (auto-lowered to 20 if too sparse). Transcriptional programs were assembled from co-expressed regulons. The resulting regulon-definition table and eigengene matrix were imported into R for downstream association analyses.

### 2.8. Identification of Mechanobiology- and Immune-Associated Regulons

Regulon target genes were tested for overlap with each curated mechanobiology and immune panel using one-sided hypergeometric tests (phyper(k − 1, sig_n, universe − sig_n, reg_n, lower.tail = FALSE)) with Benjamini–Hochberg FDR; regulons at FDR < 0.05 were retained. In parallel, regulon eigengenes were correlated with mechanobiology and immune scores by Spearman correlation. A matrix–regulon–immune network was built from significant regulon–immune associations, with each regulon annotated by its dominant mechanobiology axis. Regulon activity and state-level mechanobiology scores were compared across transcriptional states (e.g., integrin-axis vs matrix-axis states; State 0/5/8 and 0/2/7 contrasts), and state survival associations were evaluated by Cox and Kaplan–Meier.

### 2.9. Fibrosis, CAF, and ECM-Disentanglement Analyses

To distinguish structural ECM remodeling from CAF abundance, non-overlapping ECM and CAF gene sets were defined and explicitly checked for shared genes. Each score was calculated as the mean of gene-wise z-scored expression values. Independent associations of ECM and CAF programs with immune endpoints were assessed using mutual partial Spearman correlation, with each program adjusted for the other.

Linear model variance partitioning was used to estimate the variance explained by ECM-only, CAF-only, combined ECM-plus-CAF models, and their shared component. Tumor immune phenotypes were classified from infiltration and exclusion features, and their distribution across ECM score strata was compared statistically.

Gene set overlap was quantified with the Jaccard index, defined as the number of shared genes divided by the number of genes in the union of the two sets, computed pairwise across six signatures: the clean ECM_Structural and CAF_Identity sets, the original ECM_Stiffness_Core and Matrix_Remodeling axes, and the canonical myCAF and iCAF signatures. The resulting symmetric overlap matrix is reported in Supplementary Table S2. ECM_Structural and CAF_Identity shared no genes (Jaccard = 0), confirming that the mutual partial correlation and variance partitioning analyses are not affected by shared gene circularity. Residual overlap between the original signatures was retained for transparency rather than removed: Matrix_Remodeling shares ACTA2 with myCAF (Jaccard = 0.07), ECM_Stiffness_Core and ECM_Structural share POSTN with myCAF (Jaccard = 0.06), CAF_Identity shares FAP and PDGFRB with myCAF (Jaccard = 0.11) and CXCL12, CXCL14 and PDGFRA with iCAF (Jaccard = 0.18). Because Matrix_Remodeling additionally contains TGFB1 and ACTA2, its associations with the TGFβ-response score and with myofibroblast features are partly definitional for those two genes; these associations were therefore re-evaluated after removing TGFB1 and ACTA2 from the signature as a sensitivity analysis.

### 2.10. Survival Analyses

Survival analyses were performed for overall survival (OS) and progression-free interval (PFI). Overall survival was available for 536 tumors and progression-free interval for 515. Cox proportional hazards models were fit using standardized continuous mechanobiology, immune, regulon, and RPPA-derived scores, or categorical transcriptional state, as appropriate; continuous scores were standardized to unit standard deviation before modeling, so that hazard ratios are reported per standard deviation. Both univariate and stage-adjusted models were evaluated. Hazard ratios, 95% confidence intervals, and Wald *p* values are reported. The number of samples and events for each model is given in the corresponding figure legend or in Supplementary Table S3. Kaplan–Meier curves were estimated using survfit, and group differences were assessed using the log-rank test. For FAK/SRC-focused analyses, the SRC–FAK activation ratio and FAK/SRC quadrant strata were additionally evaluated.

### 2.11. Statistical Analysis

Unless otherwise specified, associations between continuous scores and ordered tumor stage were assessed using the Kruskal–Wallis test. Pairwise post hoc comparisons were performed using Dunn’s test with Benjamini–Hochberg correction. Continuous associations among mechanobiology scores, immune features, regulon eigengenes, and RPPA scores were quantified using Spearman rank correlation, with multiple-testing correction applied within the relevant test family.

Stage-controlled partial Spearman correlations were used to evaluate whether mechano-immune associations were independent of AJCC stage. Categorical distributions were compared using the χ^2^ test. All reported false-discovery rates were calculated using the Benjamini–Hochberg procedure.

#### 2.11.1. Software and Reproducibility

Analyses were performed in R (version 4.3.3) and Python (version 3.12.2). R packages included TCGAbiolinks (v2.32), edgeR (v4.2), GSVA (v2.0.7), GSEABase (v1.66), msigdbr (v7.5.1), tidyverse (v2.0.0), data.table (v1.15), survival (v3.7), ppcor (v1.1), broom (v1.0.6), rstatix (v0.7.2), pheatmap (v1.0.12), ggplot2 (v3.5.1), and ggrepel (v0.9.5). Python analyses used MINER3 together with numpy (v1.26), pandas (v2.2), and matplotlib (v3.8).

#### 2.11.2. Data and Code Availability

This study used publicly available TCGA-KIRC data from the Genomic Data Commons and UCSC Xena, the Thorsson et al. PanImmune resource, and MSigDB-derived gene sets. Analysis code and processed intermediate files should be made available on request.

## 3. Results

### 3.1. Stage-Associated Matrix Remodeling Reveals Axis-Specific Mechanobiology in ccRCC

To determine whether mechanical and matrix-associated transcriptional programs change during ccRCC progression, we first quantified a curated collection of matrix and mechano-related gene sets (Supplementary Table S1) using ssGSEA in tumor-only TCGA-KIRC RNA-seq data (Supplementary Figure S1).

The strongest stage-associated module was Fluid_Shear_Stress (Kruskal–Wallis *p* = 2.02 × 10 ^−8^, FDR = 1.61 × 10^−7^), followed by YAP_TAZ_Activity (*p* = 7.64 × 10^−6^, FDR = 3.06 × 10^−5^), Adhesion_Cytoskeleton (*p* = 1.62 × 10^−4^, FDR = 4.33 × 10^−4^), Solid_Mechanical_Stimulus (*p* = 8.83 × 10^−4^, FDR = 1.77 × 10^−3^), and ECM_Deposition (*p* = 1.26 × 10^−3^, FDR = 2.02 × 10^−3^). Hippo_Active also showed a weaker but significant stage association (*p* = 2.996 × 10^−2^, FDR = 3.99 × 10^−2^), whereas ECM_Remodeling showed a borderline association after multiple-testing correction (*p* = 4.43 × 10^−2^, FDR = 5.06 × 10^−2^). In contrast, the aggregate Integrin_FAK_RhoA module was not significantly different across stages (*p* = 0.208, FDR = 0.208), suggesting that integrin-related mechanotransduction changes were not captured by a single uniform module-level trend.

At the level of individual signatures, stage-associated changes were particularly evident in matrix organization and remodeling pathways. Collagen formation was significantly associated with stage (FDR = 5.32 × 10^−4^), as were collagen fibril organization (FDR = 5.56 × 10^−4^), epithelial–mesenchymal transition (FDR = 1.01 × 10^−3^), core matrisome activity (FDR = 4.97 × 10^−3^), degradation of the extracellular matrix (FDR = 9.14 × 10^−3^), and extracellular matrix organization (FDR = 1.44 × 10^−2^). Stage-wise boxplots and average stage heatmaps indicated that these ECM-related programs were preferentially elevated in advanced-stage tumors, particularly Stage III and Stage IV, supporting the presence of a stage-associated matrix-remodeling phenotype (Supplementary Figure S1). Because ccRCC is not necessarily a uniformly stiff tumor at the bulk tissue level, these findings are best interpreted as increased stiffness-associated matrix and ECM remodeling transcriptional activity rather than direct evidence of increased physical tissue stiffness.

The adhesion and cytoskeletal compartment showed a more complex pattern. Stress fiber assembly was one of the most stage-associated signatures overall (FDR = 3.38 × 10^−6^), and focal adhesion assembly was also significantly stage-associated (FDR = 4.90 × 10^−5^). However, the stage-wise distributions indicated that stress fiber, FAK, and RhoA-related signatures tended to decrease rather than increase with advanced stage. Consistently, both PID_FAK_PATHWAY and PID_RHOA_PATHWAY were significantly associated with stage (both FDR = 1.05 × 10^−4^), but their stage patterns diverged from those of the ECM deposition/remodeling programs. These observations might suggest that ccRCC progression is accompanied by selective matrix remodeling rather than global activation of canonical actomyosin and focal adhesion programs.

Integrin pathway signatures further supported this axis-specific behavior. Although the combined Integrin_FAK_RhoA module was not significant at the aggregate level, several individual integrin pathways were stage-associated, including PID_INTEGRIN2_PATHWAY (FDR = 1.05 × 10^−4^), PID_INTEGRIN3_PATHWAY (FDR = 1.71 × 10^−2^), PID_INTEGRIN5_PATHWAY (FDR = 2.18 × 10^−2^), and PID_INTEGRIN1_PATHWAY (FDR = 3.72 × 10^−2^). In particular, selected integrin programs showed higher activity in advanced-stage tumors, whereas FAK/RhoA and stress fiber-associated signatures showed an opposite or attenuated pattern. This divergence suggests that matrix–integrin remodeling in ccRCC may not follow a simple linear model of ECM deposition, leading to uniform FAK/RhoA/actomyosin activation.

Together, the analyses show that progression in ccRCC is associated with selective remodeling of matrix and mechanical response transcriptional programs. The dominant signal was an increase in collagen organization, ECM deposition, and matrix remodeling in advanced-stage tumors, accompanied by heterogeneous integrin pathway behavior and relative attenuation of canonical FAK/RhoA/stress fiber signatures. These results motivated the subsequent use of smaller, axis-specific mechanobiology signatures focused on ECM stiffness-associated matrix organization, integrin mechanosensing, and matrix remodeling, rather than relying solely on broad heterogeneous ssGSEA modules.

### 3.2. Focused Matrix and Integrin Signatures Refine Stage-Associated Matrix and Mechanosensing Programs in ccRCC

**Figure 1:**
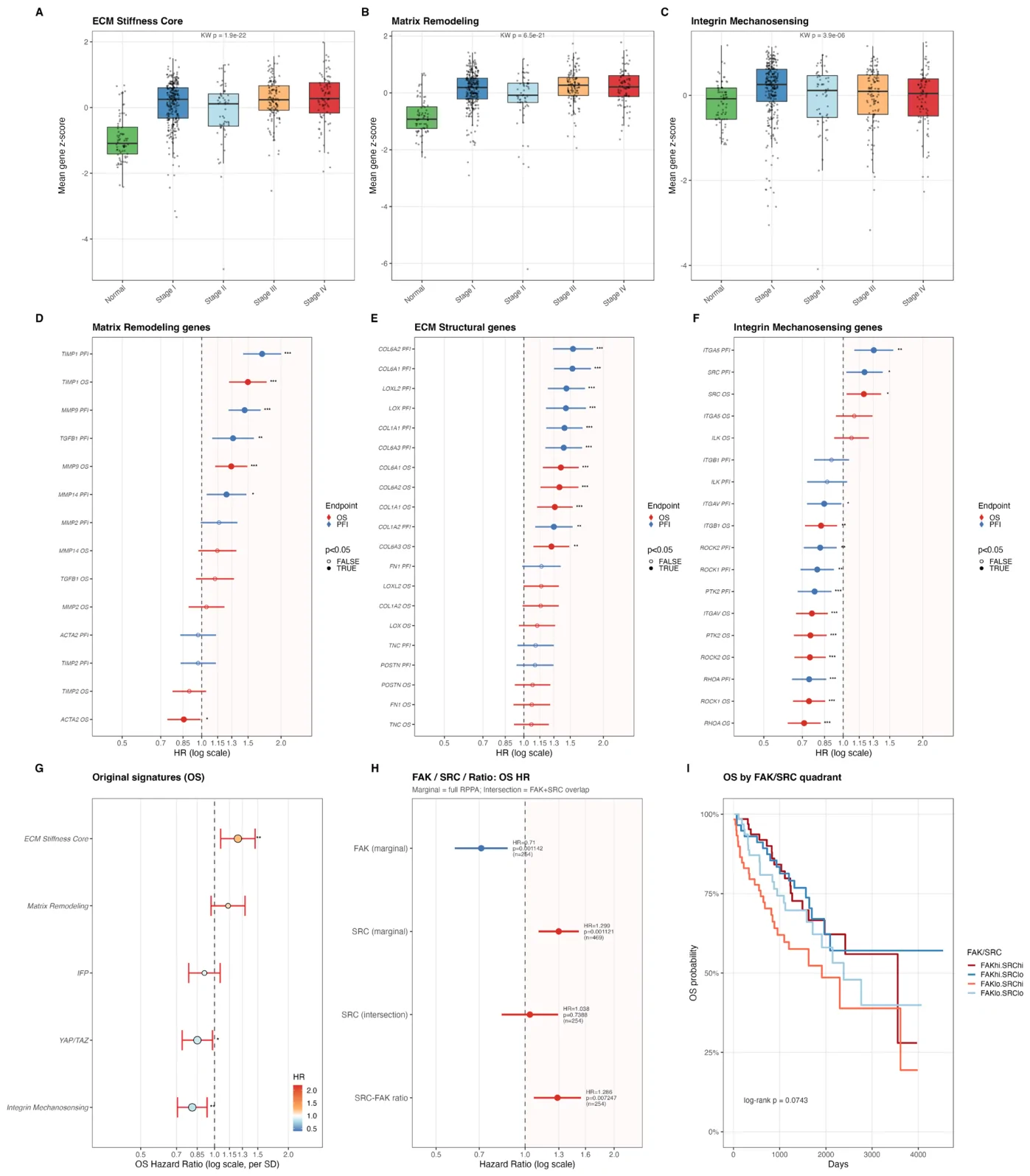
Focused matrix, integrin, and FAK/SRC signaling analyses in TCGA-KIRC. (**A**–**C**) Signature score (mean gene-wise z-scores) distributions of ECM_Stiffness_Core, Matrix_Remodeling, and Integrin_Mechanosensing signatures across normal kidney and TCGA-KIRC tumor stages. Boxplots show median and interquartile range; individual points represent samples. Kruskal–Wallis *p* values are shown in each panel. (**D**–**F**) Univariable Cox forest plots for genes included in the Matrix_Remodeling, ECM structural, and Integrin_Mechanosensing gene sets. Hazard ratios are shown separately for overall survival (OS) and progression-free interval (PFI). Points indicate hazard ratio estimates, horizontal lines indicate confidence intervals, and the dashed vertical line indicates HR = 1. (**G**) Univariable OS Cox forest plot for the original focused mechanobiology signatures. Hazard ratios are shown per standard deviation increase in signature score. (**H**) RPPA-based univariable Cox forest plot for FAK activation, SRC activation, SRC activation restricted to the FAK/SRC intersection sample, and the SRC–FAK activation ratio. Hazard ratios are shown per standard deviation, with points indicating estimates and horizontal lines indicating 95% confidence intervals. To avoid sampling bias, marginal FAK and SRC activation were each modeled on all tumors with that protein measured (FAK, n = 254; SRC, n = 469), whereas the intersection SRC model and the SRC–FAK ratio were restricted to the 254 tumors with both proteins and complete survival data. (**I**) Kaplan–Meier OS curves stratified by FAK/SRC activation quadrant, defined by an independent median split on FAK_activation and SRC_activation: FAK-low/SRC-low (n = 67), FAK-high/SRC-low (n = 60), FAK-low/SRC-high (n = 60), and FAK-high/SRC-high (n = 67), with FAK-low/SRC-low as the reference. The four-group log-rank *p* value is shown (*p* = 0.0743). Asterisks (*) denote statistical significance (* *p* < 0.05, ** *p* < 0.01, *** *p* < 0.001).

Since broad modules captured divergent ECM-, integrin-, cytoskeletal-, and FAK/RhoA-related programs, smaller custom signatures focus on ECM-associated matrix organization, integrin/focal adhesion mechanosensing, YAP/TAZ mechanosensing, interstitial fluid pressure, and matrix remodeling. ECM_Stiffness_Core and Matrix_Remodeling showed the most robust stage-associated differences, both remaining significant after multiple-testing correction (Figure 1A,B). Integrin_Mechanosensing showed a weaker, borderline stage association, whereas YAP_TAZ_Mechanosensing and Interstitial_Fluid_Pressure were less consistent (Figure 1C). The most stable transcriptomic signal during ccRCC progression was matrix-centered rather than uniformly distributed across all mechanosensing-related pathways.

Gene-level survival analyses supported this matrix-centered interpretation while also showing that individual matrix components were not prognostically equivalent. Within the Matrix_Remodeling set, TIMP1 and MMP9 showed concordant adverse associations with both overall survival and progression-free interval, whereas TGFB1 and MMP14 were more prominent for progression-free interval; in contrast, ACTA2 showed a protective overall survival direction (Figure 1D). Within the ECM structural set, collagen VI genes, COL1A1, LOX, and LOXL2 showed predominantly adverse associations, particularly for progression-free interval, while FN1, TNC, and POSTN showed weaker or non-significant associations in this univariate framework (Figure 1E). At the signature level, the original ECM_Stiffness_Core and Matrix_Remodeling scores were associated with adverse overall survival direction, whereas Integrin_Mechanosensing and YAP/TAZ-related scores showed lower-risk directions in the same univariate comparison (Figure 1G).

Component-level analysis of the Integrin_Mechanosensing signature helped explain why the aggregate integrin/focal adhesion score did not behave as a uniform adverse program. In univariate Cox models without stage adjustment, downstream focal adhesion and actomyosin-associated components showed lower-risk directions, including RHOA for OS (HR = 0.711, *p* = 3.46 × 10^−6^), PTK2/FAK for OS (HR = 0.751, *p* = 8.68 × 10^−5^), ROCK1 for OS (HR = 0.742, *p* = 3.36 × 10^−5^), and ROCK2 for OS (HR = 0.748, *p* = 3.09 × 10^−5^). In contrast, selected integrin/SRC-associated components showed higher-risk directions, including SRC for OS (HR = 1.197, *p* = 0.0191) and PFI (HR = 1.204, *p* = 0.0221), and ITGA5 for PFI (HR = 1.305, *p* = 0.0022). Although exploratory, these single-gene analyses suggest branch-skewed integrin signaling, in which preservation of the PTK2/FAK–RHOA–ROCK branch is associated with lower risk, whereas selected integrin/SRC-associated components are associated with higher-risk biology (Figure 1F).

Focused FAK/SRC analyses further supported a non-linear and partially decoupled focal adhesion signaling model. FAK and SRC activation were only weakly correlated (Spearman ρ = 0.111), indicating that these canonical focal adhesion components did not behave as a single uniformly activated axis. In univariate Cox models, FAK activation was associated with better overall survival (HR = 0.71, *p* = 0.0011), whereas SRC activation in the full RPPA sample was associated with worse overall survival (HR = 1.30, *p* = 0.0011). However, when SRC was evaluated in the FAK/SRC intersection sample, its association was attenuated and was no longer significant (HR = 1.04, *p* = 0.739). In contrast, SRC dominance relative to FAK, captured by the SRC–FAK activation ratio, remained associated with worse overall survival (HR = 1.29, *p* = 0.0072) (Figure 1H). Consistently, quadrant-based survival analysis suggested a worse outcome for FAK-low/SRC-high tumors and a more favorable pattern for FAK-high/SRC-low tumors, although the four-group log-rank comparison was borderline (*p* = 0.0743) (Figure 1I). Together, these findings suggest that adverse focal adhesion signaling in ccRCC is better captured by SRC–FAK imbalance or decoupling than by uniform activation of the canonical FAK/SRC axis.

These focused RNA and RPPA analyses support three conclusions. First, stage-associated mechanobiology in ccRCC is dominated by matrix-centered transcriptional remodeling, particularly ECM-associated matrix organization and collagen/LOX-related architecture. Second, the integrin/focal adhesion axis appears branch-skewed: canonical PTK2/FAK–RHOA–ROCK and actomyosin-associated components are attenuated in the progression-associated setting and are linked to lower-risk directions, whereas selected ITGA5/ILK/SRC-associated components show higher-risk directions. Third, RPPA-based analyses support this decoupled model at the protein-signaling level, with FAK activation associated with better survival, SRC dominance relative to FAK associated with worse survival, and the FAK-low/SRC-high quadrant showing the most adverse trend. Thus, adverse mechanobiology in ccRCC is better described as matrix-centered remodeling with branch-skewed integrin signaling and SRC–FAK imbalance, rather than as uniform activation of a canonical stiffness–FAK/RhoA mechanotransduction axis.

### 3.3. Matrix and Integrin-Related Programs Are Coupled to Immune-Suppressive Remodeling in ccRCC

We next asked whether the matrix-associated and integrin-linked programs were coupled to immune-regulatory features in TCGA-KIRC tumors. Rather than surveying the entire immune landscape, we focused on predefined suppressive and effector programs relevant to ccRCC, including TGFβ/stromal activation, CAF/fibroblast abundance, adenosine metabolism, checkpoint/exhaustion markers, Treg-associated genes, myeloid features, and cytotoxic activity (Supplementary Figure S2).

**Figure 2:**
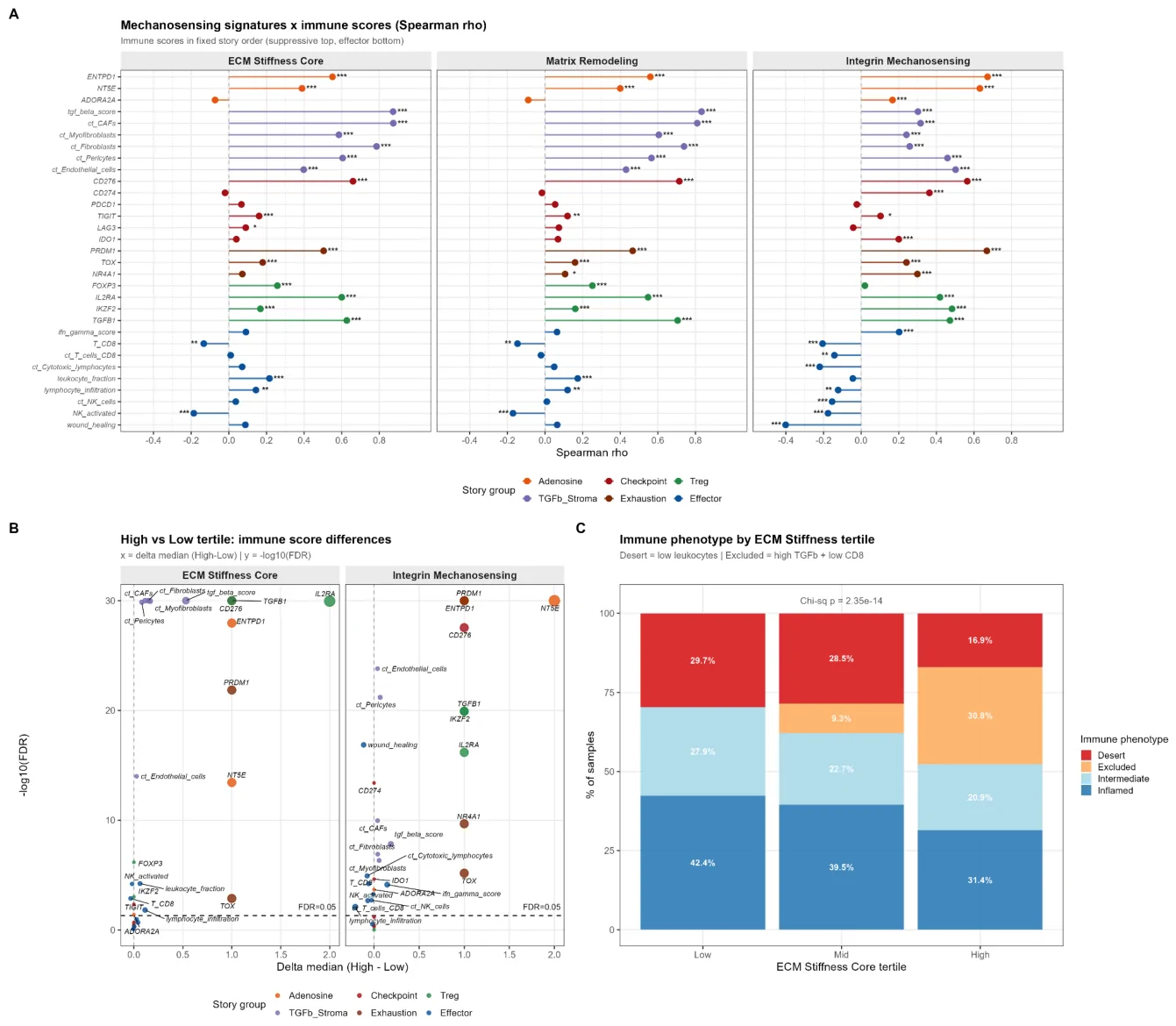
Associations between focused mechanobiology signatures and immune-regulatory features. (**A**) Spearman correlation plots between ECM_Stiffness_Core, Matrix_Remodeling, or Integrin_Mechanosensing scores and predefined immune-regulatory scores or marker genes. Points indicate Spearman correlation coefficients. Immune features are grouped by story category, including adenosine, checkpoint, Treg, TGFβ/stroma, exhaustion, and effector features. Asterisks indicate statistical significance (* *p* < 0.05, ** *p* < 0.01, *** *p* < 0.001). (**B**) High-versus-low tertile comparison plots for immune features across ECM_Stiffness_Core and Integrin_Mechanosensing groups. The x-axis shows median score difference between high and low signature tertiles, and the y-axis shows −log10(FDR). Point color indicates story group and point size indicates effect magnitude. The dashed horizontal line marks FDR = 0.05. (**C**) Stacked bar plot showing immune phenotype distribution across ECM_Stiffness_Core tertiles. Bar segments indicate the percentage of samples classified as inflamed, intermediate, excluded, or desert. Chi-square *p* value is shown.

Across tumors, ECM_Stiffness_Core and Matrix_Remodeling showed the strongest continuous associations with stromal and suppressive immune programs. ECM_Stiffness_Core was highly correlated with CAF abundance (Spearman ρ = 0.875, FDR = 1.77 × 10 ^−167^), TGFβ score (ρ = 0.873, FDR = 2.46 × 10^−160^), and fibroblast abundance (ρ = 0.788, FDR = 1.15 × 10^−112^). Matrix_Remodeling showed a similar pattern, including strong correlations with TGFβ score (ρ = 0.829, FDR = 4.04 × 10^−130^), CAF abundance (ρ = 0.812, FDR = 1.15 × 10^−124^), and fibroblast abundance (ρ = 0.740, FDR = 3.75 × 10^−92^) (Figure 2A). Because the Matrix_Remodeling signature contains TGFB1, we repeated this analysis with TGFB1 removed. The correlation was essentially unchanged (ρ = 0.832 with and without TGFB1, n = 517), indicating that the association is carried by the matrix-remodeling components (MMPs, TIMPs) rather than by ligand transcript abundance alone. Similarly, removing shared ACTA2 from the myofibroblast panel yielded a nearly identical correlation (ρ = 0.615 vs. 0.603). The equivalent CAF comparison behaved the same way (ρ = 0.738 vs. 0.728), and the fibroblast panel, which shares no genes with Matrix_Remodeling, gave a correlation of comparable magnitude (ρ = 0.679). These findings indicate that ECM-associated matrix architecture and remodeling are tightly coupled to a stromal/TGFβ-rich microenvironment.

Immune-regulatory markers showed axis-specific differences. CD276/B7-H3 was strongly associated with both Matrix_Remodeling (ρ = 0.713, FDR = 1.58 × 10^−82^) and ECM_Stiffness_Core (ρ = 0.659, FDR = 1.97 × 10^−66^), supporting a close relationship between matrix remodeling and checkpoint-like stromal immune suppression. By contrast, Integrin_Mechanosensing was distinguished less by broad stromal abundance and more by association with adenosine- and dysfunction-related immune features. In particular, ENTPD1/CD39 correlated with Integrin_Mechanosensing (ρ = 0.669, FDR = 3.20 × 10^−69^), NT5E/CD73 showed a similarly strong integrin-linked pattern, and PRDM1, a marker associated with dysfunctional/exhausted immune states, was also strongly associated (ρ = 0.665, FDR = 4.59 × 10^−68^).

This distinction was also evident in the high-versus-low effect size comparison plots (Figure 2B). Tumors with high ECM_Stiffness_Core or Matrix_Remodeling scores showed the largest median increases in stromal features, including CAFs, fibroblasts, pericytes, myofibroblasts, and TGFβ-related programs, together with marked enrichment of suppressive immune markers such as CD276, TGFB1, ENTPD1, NT5E, PRDM1, and IL2RA. In contrast, high Integrin_Mechanosensing tumors showed a comparatively stronger shift toward adenosine-related (ENTPD1, NT5E) and immune dysfunction/Treg-associated features (PRDM1, TOX, NR4A1, IKZF2, IL2RA), whereas cytotoxic endpoints remained closer to the null or showed substantially smaller effect sizes. These results reinforce the conclusion that matrix architecture/remodeling primarily tracks a stromal suppressive niche, whereas the integrin-linked program is more selectively aligned with adenosine- and dysfunction-associated immune suppression.

To determine whether these relationships were simply explained by tumor stage, we performed stage-controlled partial correlation analyses using three composite immune programs: adenosine score, total suppression, and cytotoxic activity. The total suppression composite remained strongly associated with ECM_Stiffness_Core (partial ρ = 0.903, FDR = 1.69 × 10^−196^) and Matrix_Remodeling (partial ρ = 0.875, FDR = 8.74 × 10^−169^), and more moderately with Integrin_Mechanosensing (partial ρ = 0.506, FDR = 7.83 × 10^−36^). By contrast, adenosine signaling was most strongly associated with Integrin_Mechanosensing (partial ρ = 0.692, FDR = 7.00 × 10^−77^), with additional but weaker associations for ECM_Stiffness_Core (partial ρ = 0.519, FDR = 9.85 × 10^−38^) and Matrix_Remodeling (partial ρ = 0.489, FDR = 2.63 × 10^−33^). Correlations with cytotoxic activity were significant but consistently weaker for all three axes (partial ρ = 0.170–0.216). Together, these findings indicate that matrix architecture/remodeling and integrin-linked programs in ccRCC are coupled predominantly to immune-suppressive remodeling rather than to cytotoxic immune activation alone.

Together, these findings support the notion that matrix remodeling and integrin-associated programs are coupled with immune dysfunction and stromal immune suppression in ccRCC, rather than with cytotoxic immune activity alone.

### 3.4. Matrix Remodeling Retains Immune-Suppressive Information Beyond CAF Abundance and Recapitulates a Fibrosis-like Immune Phenotype

Because ECM remodeling and CAF abundance were strongly coupled in the preceding analyses, we next asked whether the matrix–immune associations simply reflected fibroblast enrichment. This was important because many CAF and matrix signatures share structural ECM genes, including collagens, fibronectin, POSTN, and ACTA2. To address this, we generated gene-overlap-cleaned ECM structural and CAF identity scores and repeated the immune correlation analyses using mutual adjustment and variance partitioning approaches.

Even after removing shared genes, clean ECM structural and clean CAF identity scores remained strongly correlated across tumors (Spearman ρ = 0.852, *p* = 5.01 × 10^−152^), indicating that matrix remodeling and CAF abundance represent biologically co-varying stromal programs rather than only a gene set overlap artifact. In mutual partial correlation analyses across eight immune endpoints, ECM was the stronger independent correlate for five endpoints, whereas three were ambiguous and none were CAF-dominant. This pattern was most evident for CD276/B7-H3 and TGFβ signaling. CD276 retained a substantial ECM association after controlling for CAF identity (raw ρ = 0.660; partial ρ = 0.416), whereas the CAF association nearly disappeared after controlling for ECM structure (raw ρ = 0.563; partial ρ = 0.002). Similarly, TGFβ score remained strongly associated with ECM after CAF adjustment (raw ρ = 0.873; partial ρ = 0.663), while the independent CAF signal was markedly reduced (raw ρ = 0.760; partial ρ = 0.066). ECM-dominant patterns were also observed for ENTPD1 and PRDM1, whereas cytotoxic T-cell-related endpoints were weak or ambiguous.

Variance partitioning analyses supported the same conclusion. For CD276, ECM alone explained more variance than CAF alone (R^2^ = 0.424 versus 0.370), with a large shared component (R^2^_shared = 0.352), consistent with tightly coupled but non-identical stromal programs. For TGFβ score, ECM alone again explained more variance than CAF alone (R^2^ = 0.774 versus 0.562), and adding CAF to ECM provided little additional explanatory gain. Thus, although CAF-rich tumors are also matrix-remodeled tumors, key suppressive immune features are more closely aligned with ECM structural remodeling than with CAF abundance alone.

We next tested whether a fibrosis-associated ECM remodeling axis recapitulated the same immune-suppressive phenotype. In stage-controlled partial correlation analyses, the fibrosis-associated ECM score remained strongly associated with TGFβ score (partial ρ = 0.875), CAF abundance (partial ρ = 0.872), fibroblast abundance (partial ρ = 0.784), CD276/B7-H3 (partial ρ = 0.655), TGFB1 expression (partial ρ = 0.620), myofibroblast abundance (partial ρ = 0.602), IL2RA (partial ρ = 0.588), ENTPD1/CD39 (partial ρ = 0.569), and PRDM1 (partial ρ = 0.509). Immune phenotype classification further showed that ECM-high tumors were not simply immune-desert tumors; rather, they were enriched for both inflamed and excluded phenotypes, with 32.9% classified as inflamed and 30.0% as excluded (Figure 2C). The distribution of immune phenotypes differed significantly across ECM tertiles (χ^2^ *p* = 3.74 × 10^−13^), supporting a shift toward a stromal/fibrotic immune-remodeled state rather than a uniform absence of immune cells.

Together, these analyses argue that the mechano-immune phenotype identified in ccRCC cannot be reduced to generic CAF abundance. CAF enrichment and matrix remodeling are tightly linked, but ECM structural remodeling retains independent associations with key suppressive immune features, particularly CD276/B7-H3, TGFβ signaling, ENTPD1/CD39, and PRDM1. The fibrosis-associated ECM analysis further supports this interpretation by showing that fibrotic matrix remodeling recapitulates a TGFβ/CAF/myofibroblast-rich and immune-suppressive phenotype. Thus, remodeled ECM appears to carry immune-regulatory information beyond fibroblast abundance alone, supporting its role as a defining component of the suppressive stromal niche in ccRCC.

### 3.5. Network-Inferred Transcriptional States Separate Canonical Integrin Mechanosensing from Matrix-Remodeling-Associated Progression Risk

To determine whether mechanobiology programs were embedded within broader regulatory contexts, we next analyzed transcriptional states inferred using MINER (Mechanistic Inference of Node-Edge Relationships). MINER reconstructs transcriptional regulatory programs from gene-expression data by defining transcription factor-linked co-regulated gene modules, referred to here as regulons, and summarizing their activity as regulon eigengenes. Tumors can then be grouped into transcriptional states based on shared regulon activity patterns. We used these network-inferred states to ask whether canonical integrin-associated mechanosensing and matrix remodeling defined distinct transcriptional contexts with different clinical implications.

**Figure 3:**
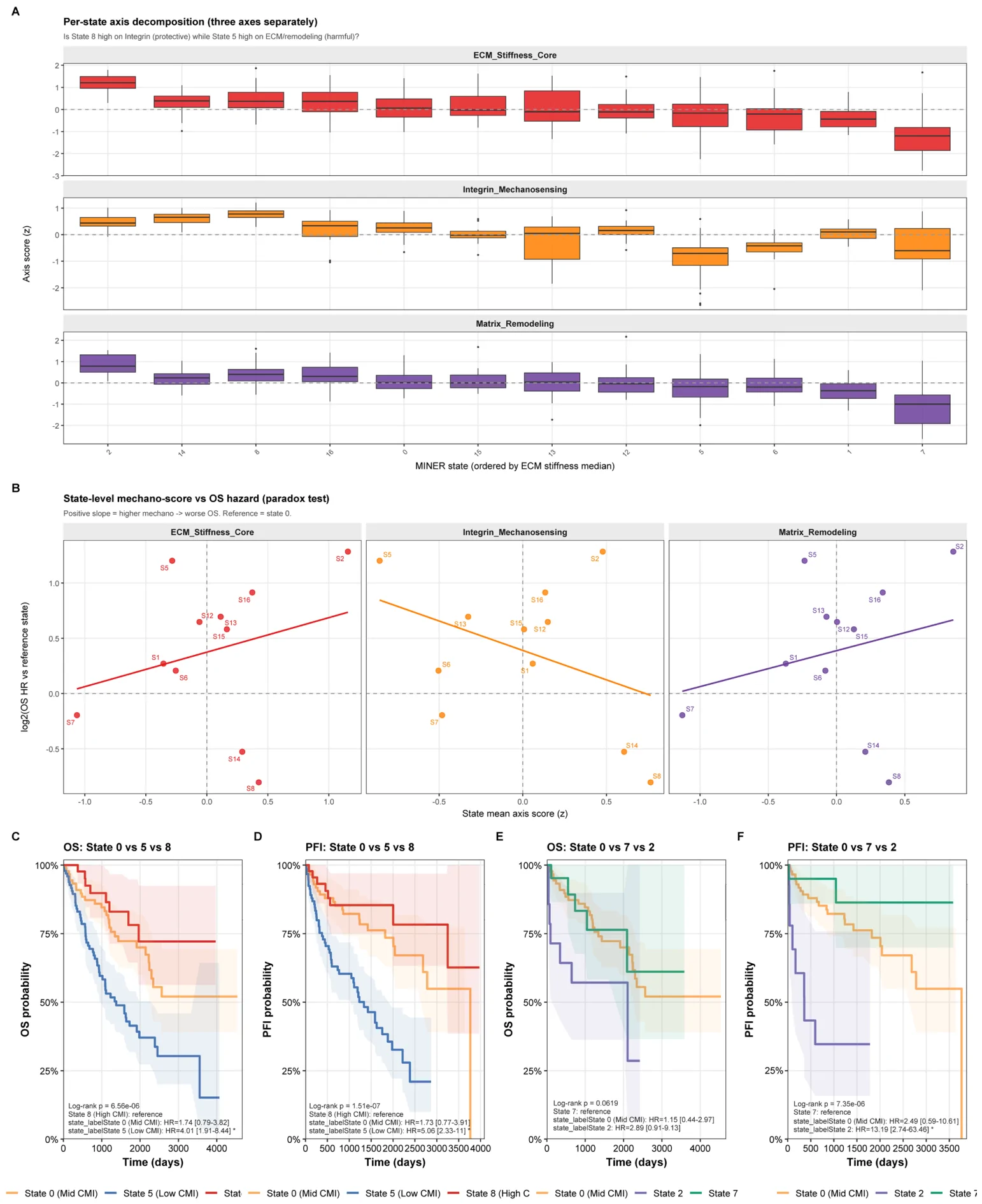
MINER transcriptional states, mechanobiology scores, and survival associations. (**A**) Per-state distributions of ECM_Stiffness_Core, Integrin_Mechanosensing, and Matrix_Remodeling scores across MINER transcriptional states. States are ordered by ECM_Stiffness_Core median. Boxplots show median and interquartile range. The dashed horizontal line indicates a mean axis z-score of zero (**B**) State-level mechanobiology score versus OS hazard plot. The x-axis shows mean state-level mechanobiology score, and the y-axis shows log2 OS hazard ratio relative to the reference state. Each point represents a MINER state; linear trend lines are shown for each signature. The dashed horizontal line indicates no change in hazard relative to the reference state (log2 HR = 0), and the dashed vertical line indicates a mean state-level axis score of zero. (**C**,**D**) Kaplan–Meier OS and PFI curves for States 0, 5, and 8. State 8 is used as the reference group in the displayed Cox annotation. (**E**,**F**) Kaplan–Meier OS and PFI curves for States 0, 7, and 2. State 7 is used as the reference group in the displayed Cox annotation. Log-rank *p* values and hazard ratio annotations are shown within the panels. * *p* < 0.05.

We first focused on the Integrin_Mechanosensing signature, which captured a canonical focal adhesion/mechanosensing axis, including integrins, PTK2/FAK, SRC, ILK, RHOA, ROCK1, and ROCK2. Across MINER states, this axis showed marked heterogeneity. To examine this axis at the state level, we selected the two states occupying the extremes of Integrin_Mechanosensing activity: State 5, which showed the lowest activity, and State 8, which showed the highest, together with State 0 as an intermediate reference. State 0 was chosen because it was one of the most populated states with mid-range mechanosensing activity. State 5 and State 8 had mean state-level Integrin_Mechanosensing scores of −0.856 and 0.764, respectively (Figure 3A). This extreme-versus-extreme contrast was larger and more specific to the canonical integrin–FAK/SRC–RhoA mechanosensing program than the corresponding matrix-associated differences, making the State 5 versus State 8 comparison a useful model for examining focal adhesion-linked mechanosensing rather than broader ECM abundance alone. Per-state sample sizes are reported in Supplementary Table S3 (State 5, n = 97; State 0, n = 92; State 8, n = 48).

At the state level, matrix-associated programs and Integrin_Mechanosensing showed divergent relationships with overall survival risk. When state-level mean mechanobiology scores were plotted against the log2 overall survival hazard ratio relative to State 5, ECM_Stiffness_Core and Matrix_Remodeling showed positive trends, consistent with higher matrix-associated activity being linked to higher-risk state contexts. In contrast, Integrin_Mechanosensing showed a negative trend: states with higher canonical integrin/focal adhesion mechanosensing tended to have lower overall survival hazard. Notably, State 8 combined high Integrin_Mechanosensing with low OS risk relative to State 5, whereas State 2 showed high matrix-associated scores and a higher-risk direction. These state-level patterns support a separation between matrix-remodeling-associated risk and canonical integrin/focal adhesion mechanosensing, rather than a single uniform mechanobiology-risk axis (Figure 3B).

This interpretation was supported by the component-level analysis of the Integrin_Mechanosensing signature. Although the aggregate score included upstream integrin and SRC-associated components, individual genes showed divergent survival directions. In univariate Cox models, downstream canonical focal adhesion and actomyosin components were associated with lower risk, including RHOA, ROCK1, ROCK2, and PTK2/FAK, whereas selected integrin/SRC-associated components, including SRC and ITGA5, were associated with higher risk. Thus, the protective association of the aggregate Integrin_Mechanosensing score is most consistent with preservation of the PTK2/FAK–RHOA–ROCK branch rather than uniform activation of all integrin pathway components.

This state-level pattern was consistent with the survival behavior of the continuous Integrin_Mechanosensing score. Higher Integrin_Mechanosensing activity was associated with better overall survival in univariate Cox regression (HR = 0.811, *p* = 0.0032) and remained associated with better overall survival after stage adjustment (HR = 0.847, *p* = 0.0320). The progression-free interval association was also protective in the univariate model (HR = 0.864, *p* = 0.0497), but was attenuated after stage adjustment (HR = 0.959, *p* = 0.605). Thus, lower canonical integrin/focal adhesion mechanosensing was associated with worse overall survival behavior.

The State 5 versus State 8 survival contrast provided a state-level example of this pattern. In the Kaplan–Meier analysis of States 0, 5, and 8, State 8, which showed high Integrin_Mechanosensing activity, had the most favorable survival profile, whereas State 5, which showed low Integrin_Mechanosensing activity, had the poorest outcome. Using State 8 as the reference, State 5 was associated with substantially higher risk for both overall survival (HR = 4.01, 95% CI 1.91–8.44) and progression-free interval (HR = 5.06, 95% CI 2.33–11.0), while State 0 showed an intermediate, non-significant risk profile (Figure 3C,D). These findings indicate that the adverse State 5 phenotype is linked to reduced canonical integrin/focal adhesion mechanosensing, whereas State 8 retains a higher integrin/mechanosensing program and is clinically less adverse.

Matrix-centered programs were examined separately using at the extremes of matrix-associated activity: State 2, which showed the highest ECM_Stiffness_Core and Matrix_Remodeling activity, and State 7, which showed the lowest, again with State 0 as the intermediate reference (Figure 3A,B). In Kaplan–Meier analyses of States 0, 7, and 2, State 2 showed a higher-risk direction relative to State 7, particularly for progression-free interval (HR = 13.19, 95% CI 2.74–63.46; log-rank *p* = 7.35 × 10^−6^), while the overall survival contrast was weaker and borderline (HR = 2.89, 95% CI 0.91–9.13; log-rank *p* = 0.0619) (Figure 3E,F). After stage adjustment, the State 2 progression-free interval association was attenuated but remained in the higher-risk direction (HR = 1.841, 95% CI 0.868–3.904, *p* = 0.112). Because State 2 contained relatively few tumors and confidence intervals were wide, this matrix-state contrast was interpreted as exploratory but supportive of a matrix-remodeling-associated progression risk axis.

Matrix architecture/remodeling and integrin-linked programs are coupled to immune-suppressive remodeling in ccRCC, but through partly distinct immune-regulatory profiles. ECM_Stiffness_Core and Matrix_Remodeling primarily tracked a stromal/TGFβ-rich suppressive niche, whereas Integrin_Mechanosensing was more selectively aligned with adenosine- and dysfunction-associated immune features. Cytotoxic and effector programs showed weaker associations, indicating that these mechanobiology axes are linked more closely to immune suppression and stromal remodeling than to cytotoxic immune activation alone.

### 3.6. Matrix and Integrin-Linked Regulons Define State-Dependent Immune-Regulatory Architectures

A regulon was defined as a transcription factor-linked co-regulated gene module summarized by its regulon eigengene. We used this framework to ask which inferred regulatory modules connected matrix- and integrin-associated transcriptional programs to immune-regulatory targets in ccRCC.

MINER identified 1868 regulons. In the regulon-signature correlation analysis, 60 regulons were associated with matrix, integrin, YAP/TAZ, or fluid pressure-related signatures, including 27 linked most strongly to Integrin_Mechanosensing, 16 to the ECM-associated core, six to Matrix_Remodeling, nine to YAP/TAZ_Mechanosensing, and two to Interstitial_Fluid_Pressure. We then focused on the matrix–integrin–immune network, which contained 41 regulon–immune target edges involving 33 unique regulons, 24 transcription factors, and 19 immune-regulatory target genes.

**Figure 4:**
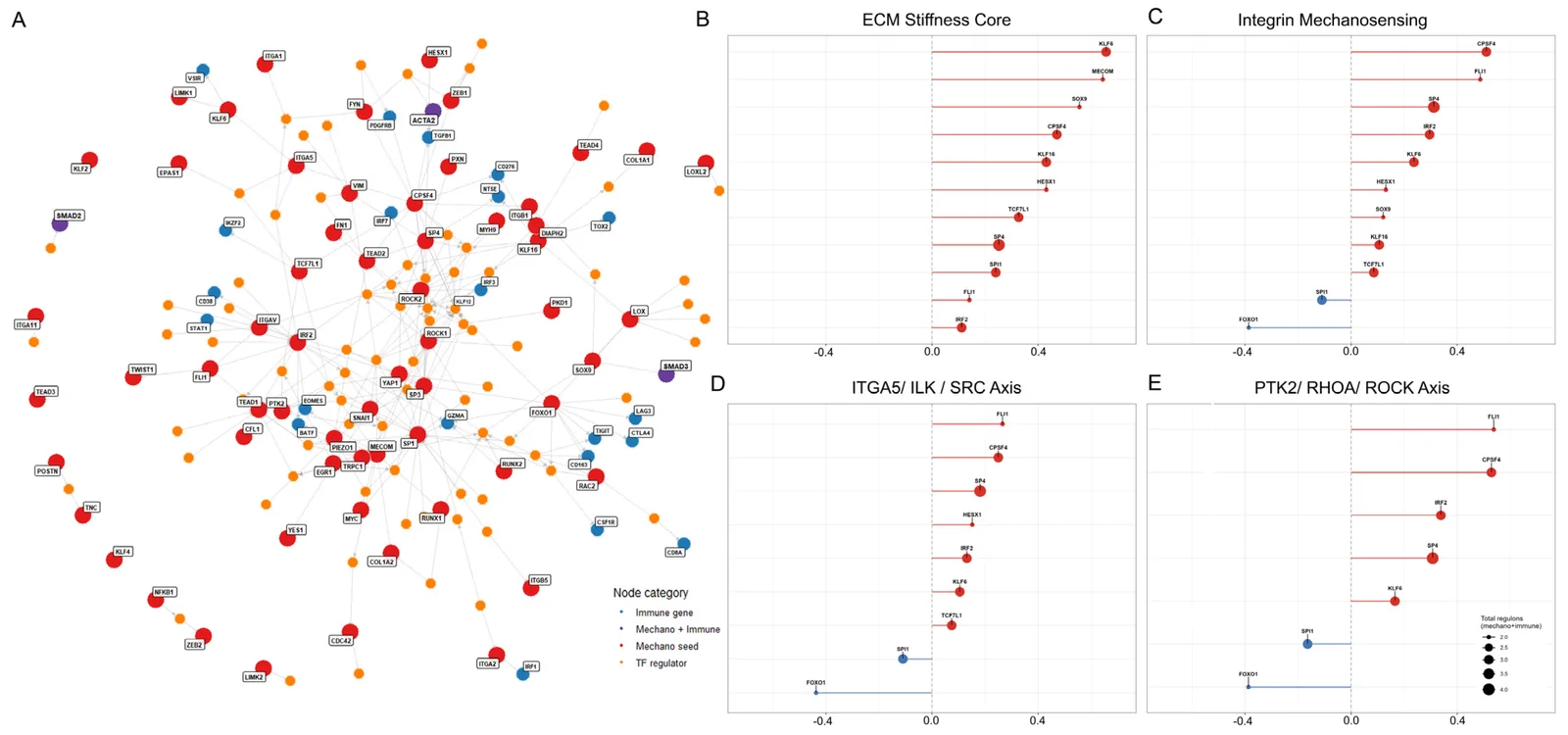
Mechano-immune-regulatory subnetwork and bridge TF analyses. (**A**) Mechano-specialized subnetwork containing mechanobiology seed genes, immune-regulatory genes, genes shared between mechanobiology and immune annotations, and inferred transcription factor regulators. Node color denotes node category: red, mechanobiology seed; blue, immune gene; purple, mechano + immune gene; orange, transcription factor regulator. Edges indicate inferred network connectivity. (**B**,**C**) Bridge TF lollipop plots for ECM_Stiffness_Core and Integrin_Mechanosensing. The x-axis shows mean Spearman correlation between bridge TF regulon activity and the indicated mechanobiology signature. Point size indicates the number of mechano-immune regulons assigned to each TF. Red points indicate positive associations and blue points indicate negative associations. (**D**,**E**) Bridge TF lollipop plots after separating Integrin_Mechanosensing into ITGA/ILK/SRC-associated and PTK2/RHOA/ROCK-associated branches.

Within this network, 24 edges were associated with the ECM-associated core, 16 with Integrin_Mechanosensing, and one with Matrix_Remodeling. The immune target groups were unevenly distributed: 18 edges mapped to TGFβ/stroma/Treg-related biology, 12 to myeloid/CAF exclusion, six to checkpoint/exhaustion, three to adenosine/metabolic suppression, one to inflammatory/dual signaling, and one to cytotoxicity (Figure 4A). Thus, the inferred network connected matrix- and integrin-linked regulons mainly to stromal, TGFβ/Treg, myeloid/CAF, checkpoint, and adenosine-associated immune regulation, rather than to cytotoxic immune activity alone. Resolving these edges by individual transcription factor showed that the TGFβ/stroma/Treg and myeloid/CAF-exclusion mechanism groups were driven by a limited set of regulon TFs, with KLF15 and CPSF4 accounting for the largest numbers of immune targets, and that most TF–mechanism pairs were dominated by the ECM_Stiffness_Core axis (Supplementary Figure S3).

Bridge TF analysis revealed that the ECM-associated and integrin-linked mechanobiology axes shared a core set of regulatory intermediates but showed distinct branch-specific preferences. Several bridge TFs, including KLF6, SOX9, FLI1, CPSF4, SP4, and IRF2, showed positive associations with both ECM-associated and integrin-linked signatures, connecting these structural programs to a broader stromal–immune-regulatory network (Figure 4B,C). However, when Integrin_Mechanosensing was separated into ITGA5/ILK/SRC-associated and PTK2/RHOA/ROCK-associated branches, this shared bridge TF architecture became branch-skewed: CPSF4, FLI1, and IRF2 showed stronger alignment with the PTK2/RHOA/ROCK branch than with the ITGA5/ILK/SRC branch (Figure 4D,E). This suggests tighter coupling between canonical focal adhesion/actomyosin mechanosensing and these immune-regulatory bridge modules. In contrast, FOXO1 and SPI1 showed negative associations with both integrin branches, suggesting that FOXO1- and SPI1-linked immune-regulatory modules may become relatively more prominent when canonical integrin/focal adhesion mechanosensing is reduced.

**Figure 5:**
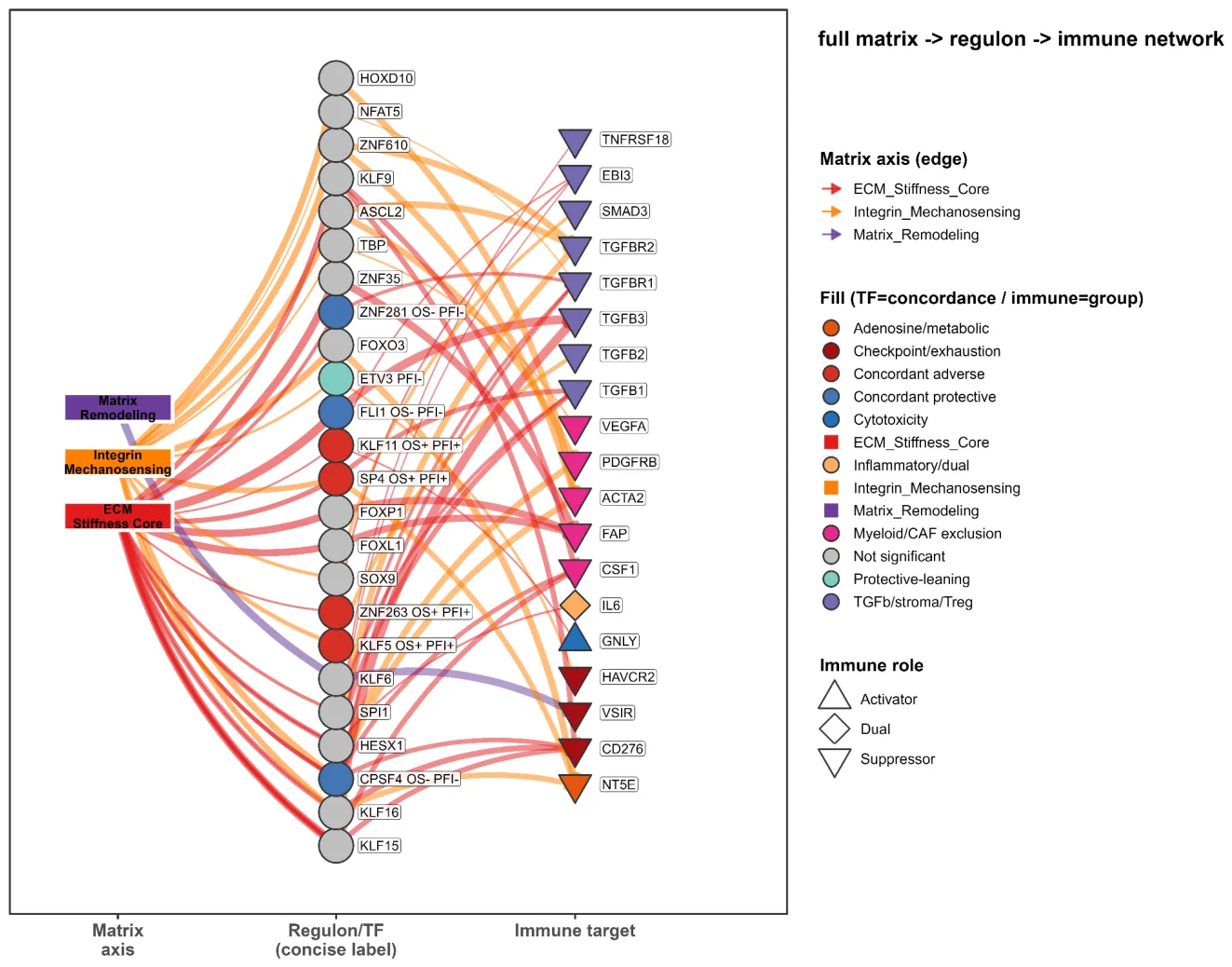
Extended matrix–regulon–immune arc network. The arc network displays inferred connections from mechanobiology axes to regulon/transcription factor modules and immune-regulatory targets. The left layer indicates the mechanobiology axis, the middle layer indicates regulon or transcription factor modules, and the right layer indicates immune-regulatory target genes. Edge color denotes the associated matrix or integrin axis: ECM_Stiffness_Core, Integrin_Mechanosensing, or Matrix_Remodeling. Edge width indicates the strength of the regulon–axis association. Node fill denotes TF concordance category or immune mechanism group, and immune target shape denotes inferred immune role: activator, dual, or suppressor. The legend defines all edge colors, node fills, and immune role shapes.

The extended matrix–regulon–immune arc network was used to map how matrix- and integrin-linked axes connected to immune-regulatory targets through inferred regulon modules (Figure 5). In this representation, the left layer denotes the mechanobiology axis, the middle layer denotes the inferred regulon or transcription factor module, and the right layer denotes the immune-regulatory target. This network showed that ECM-associated and integrin-linked axes converged on overlapping but distinct suppressive target programs. ECM-associated edges were more prominently connected to CD276/B7-H3, CSF1, TGFB1, VSIR, and stromal/myeloid-associated targets, supporting a matrix-linked checkpoint, myeloid recruitment, and TGFβ/stromal suppressive architecture. In contrast, integrin-linked edges were more prominent around NT5E/CD73 adenosine associated immune exclusion and stromal/TGFβ-receptor-associated targets such as TGFBR1, TGFBR2, SMAD3, ACTA2, and PDGFRB.

Across all MINER states, hierarchical clustering of immunosuppressive network-linked regulon activity showed that States 5 and 8 occupied distinct positions in the regulon activity landscape, with State 8 clustering apart from the bulk of remaining states (Supplementary Figure S5). We therefore examined the State 5 versus State 8 contrast in detail.

The state-level dumbbell plots added a dynamic layer to this wiring diagram by showing which regulon–immune target modules changed between biologically informative transcriptional states. In the State 5 versus State 8 comparison, the direction of change followed the integrin/mechanosensing contrast. State 8 retained higher canonical integrin/focal adhesion mechanosensing, whereas State 5 represented a low-integrin-mechanosensing, higher-risk context. For State 8, high regulons were linked to NT5E, ACTA2, PDGFRB, TGFBR2, TGFB2, HAVCR2, SMAD3, and TGFBR1, suggesting an integrin-associated regulatory context enriched for adenosine and stromal/TGFβ-receptor-related targets. In contrast, for State 5, high regulons were linked to TNFRSF18, EBI3, CD276, CSF1, TGFB1, and IL6. Thus, the shift from State 8 to State 5 can be interpreted as loss of canonical integrin/focal adhesion mechanosensing accompanied by enrichment of CD276/B7-H3 checkpoint biology, CSF1-associated myeloid recruitment, EBI3/IL35-related suppressive signaling, and TGFB1/inflammatory-stromal regulation (Supplementary Figure S4A).

The State 2 versus State 7 comparison captured a different regulatory transition centered on matrix architecture. State 7 represented a matrix-low, lower-risk context, whereas State 2 represented a collagen/LOX-rich ECM core and matrix-remodeling context. In this contrast, all available network-linked regulons showed higher activity in State 2 than in State 7, indicating coordinated activation of the matrix-linked suppressive regulatory network in the matrix-high state. The largest State 2-high differences involved CD276/CSF1-linked KLF9, CPSF4, and KLF15 modules; CD276-linked KLF16; TGFB1-linked HESX1, SP4, and SPI1 modules; and the VSIR-linked KLF6 module. Therefore, the transition from State 7 to State 2 was associated with broad enrichment of B7-H3/checkpoint, CSF1/myeloid recruitment, TGFB1/stromal-immunosuppressive, and VSIR-linked checkpoint/myeloid regulatory targets (Supplementary Figure S4B). This suggests that the matrix-high State 2 does not merely reflect elevated ECM expression, but a broader ECM-associated immune-suppressive regulatory architecture.

In simple terms, the matrix architecture axis appears to behaves as a directional suppressive amplifier, in which matrix-high tumors show coordinated enrichment of CD276/CSF1-, TGFB1-, and VSIR-linked immune-suppressive regulon activity. In parallel, integrin-linked mechanosensing appears to act as a branch balance rheostat. When the canonical PTK2/FAK–RHOA–ROCK focal adhesion/actomyosin branch is preserved, tumors show a lower-risk survival pattern and retain an integrin-linked NT5E/CD73 and stromal/TGFβ-receptor regulatory context. When this canonical branch is reduced and signaling shifts toward ITGA5/ILK/SRC-associated activity, the immune-regulatory context shifts toward CD276/CSF1/EBI3/TGFB1-centered checkpoint–myeloid–TGFβ/Treg immune exclusion, which is associated with worse clinical behavior. Thus, the adverse mechanobiology phenotype is not global activation of all matrix or integrin programs, but matrix-driven immune-suppressive remodeling combined with a branch-specific switch away from canonical FAK/RHOA mechanosensing toward SRC-skewed immune-suppressive rewiring.

## 4. Discussion

We propose a two-layer mechanobiology conceptual model in ccRCC: matrix remodeling behaves as a directional suppressive amplifier, whereas integrin-linked mechanosensing behaves as a branch balance rheostat. The dominant signal in our analyses was an ECM-associated matrix program linked to collagen/fibronectin/LOX-rich organization, CAF/fibroblast enrichment, TGFβ signaling, CD276/B7-H3, CSF1-associated myeloid biology, VSIR-linked checkpoint/myeloid regulation, and adenosine-related immune suppression. Progression was associated with coordinated enrichment of stromal and immune-suppressive programs, rather than uniform activation of all mechanobiology-related pathways. Integrin_Mechanosensing, by contrast, did not behave as a single adverse pathway. Instead, ITGA5/ILK/SRC-associated components aligned more closely with higher-risk biology, whereas the canonical PTK2/FAK–RHOA–ROCK focal adhesion/actomyosin branch showed lower-risk directions and was retained in the less adverse integrin-high state. These findings suggest that immune suppression in ccRCC is not simply a consequence of “high mechanotransduction,” Rather, they support a framework built on two partially distinct regulatory programs. The first is an ECM-dominant CD276/CSF1/TGFB1/VSIR-centered suppressive architecture. The second is an integrin branch state in which loss of canonical FAK/RHOA/ROCK mechanosensing and relative SRC-skewing shift tumors toward checkpoint–myeloid–TGFβ/Treg immune exclusion.

A key point is that this matrix program should not be interpreted as direct evidence of uniform tumor stiffening. In breast and pancreatic cancer, collagen crosslinking and ECM stiffening are well-established features of desmoplastic progression, but ECM mechanics vary substantially across tumor types and tissue contexts [13,14]. ccRCC is especially complex because it is vascular, lipid-rich, and spatially heterogeneous; available tissue and cell-level mechanical studies do not support a simple assumption that RCC is uniformly stiffer than non-tumor kidney [17,18]. Therefore, our ECM_Stiffness_Core should be read as a stiffness-associated ECM transcriptional program, not as a direct measurement of elastic modulus. The biologically relevant process may be local fibrotic matrix organization rather than global stiffness: collagen, fibronectin, and LOX-family remodeling can create crosslinked intratumoral niches that support integrin signaling, stromal activation, and immune restriction without requiring the entire tumor mass to become mechanically rigid [13].

This interpretation places ccRCC within the broader literature on ECM-mediated immune resistance while also distinguishing it from classical desmoplastic tumors. A remodeled ECM can regulate antitumor immunity by limiting immune cell movement, altering growth factor availability, activating CAF programs, and shaping checkpoint and myeloid signaling [14]. Stromal TGFβ has been shown to promote T-cell exclusion and resistance to PD-L1 blockade in other tumor contexts, supporting the general concept that matrix-rich stromal programs can suppress effective antitumor immunity [19,20]. Our findings therefore suggest a more specific model: matrix-remodeled ccRCC may remain immune-infiltrated, but the transcriptional profile is redirected toward a stromal, CAF/TGFβ, CD276/B7-H3, CSF1/myeloid, VSIR, and adenosine-associated suppressive phenotype that could contribute to immune dysfunction or exclusion rather than cytotoxic immunity. This is consistent with recent ccRCC spatial evidence that POSTN-positive CAF and CCL3-positive macrophage interactions define immune-excluded, checkpoint-high regions, including CD276-enriched niches [26].

The CAF/ECM analysis further suggests that the matrix-associated immune phenotype is not explained only by fibroblast abundance. This distinction matters because CAFs are major producers of collagen, fibronectin, periostin, and matrix-remodeling enzymes; therefore, ECM and CAF signatures are expected to overlap. Nevertheless, the persistence of ECM-associated immune correlations after adjustment for CAF identity indicates that matrix organization carries information beyond generic CAF quantity. We interpret this cautiously: the result does not fully separate ECM from CAF biology, but it indicates that collagen and matrix organization programs capture a functionally relevant stromal state. This aligns with current CAF models in which CAF identity, ECM composition, TGFβ signaling, and immune suppression are interdependent but not interchangeable features of the tumor microenvironment [27].

The integrin findings refine the model further and may be the most important mechanistic distinction in the study. Integrins transduce ECM cues via FAK, SRC, ILK, Rho-family GTPases, and actomyosin regulators, but these downstream branches do not necessarily carry the same biological meaning [24,28]. ITGA5 encodes the α5 subunit of the fibronectin receptor α5β1, a major interface between fibronectin-rich matrix and tumor cell adhesion, invasion, and survival signaling [29]. ILK has also been implicated in renal cancer invasion and metastasis, supporting the plausibility that an ITGA5/ILK-associated program reflects adverse matrix-adaptive signaling [30]. SRC provides another adverse branch because it integrates adhesion and growth-factor signaling and can promote invasion, plasticity, and survival [31]. By contrast, the relatively lower-risk direction of PTK2/FAK–RHOA–ROCK components suggests that preserved canonical focal adhesion/cytoskeletal mechanosensing is not intrinsically adverse in ccRCC. The underrepresentation of this branch in ccRCC may reflect a loss of a more organized adhesion/cytoskeletal state and a shift toward SRC/ILK-skewed matrix adaptation [32].

This conceptual branch-skewed integrin model may also help explain immune resistance more precisely. RHOA/ROCK signaling is not only a tumor cell contractility pathway; it also regulates migration, polarity, and immune synapse-related behavior in T- and B-cells, and cytoskeletal control is central to immune cell trafficking and function [33]. Tumors with reduced representation of the RHOA/ROCK-associated focal adhesion branch may reflect a broader shift in tissue organization. This shift could affect how immune cells enter, move through, and interact within the tumor microenvironment. In our data, this concept is consistent with the distinction between matrix-dominant immune suppression and integrin-linked immune remodeling. Matrix organization was most strongly aligned with CAF/TGFβ/CD276/CSF1-like suppression, whereas the integrin axis showed stronger relationships with adenosine-related immune regulation. This suggests that different mechanical programs may predict different immune-resistance mechanisms rather than a single global “mechanosuppressive” state.

The MINER state and regulon analyses provide a transcriptional-state framework for this interpretation. State-level analyses showed that high integrin/focal adhesion mechanosensing was not automatically adverse, whereas matrix-high contexts were more closely aligned with progression risk direction. At the regulon level, matrix- and integrin-linked modules converged on immune-regulatory targets, but the strongest adverse architecture involved CD276-, CSF1-, and TGFB1-linked programs. It suggests that ccRCC immune resistance may be organized through regulatory states that connect matrix remodeling to specific suppressive circuits: B7-H3/checkpoint biology, myeloid recruitment, TGFβ/stromal signaling, and adenosine metabolism. These are not interchangeable pathways, and this separation may be useful for developing and testing mechanism-informed combination strategies.

The translational implication is that matrix and integrin programs may help identify immune-resistant mechanisms that are not captured by immune infiltration alone. A matrix-high, TGFB1/CD276/CSF1-rich tumor may require a different support therapy than an integrin/adenosine-skewed tumor. For matrix-dominant immune resistance, the most rational combinations would involve checkpoint blockade with stromal TGFβ modulation, B7-H3/CD276 targeting, CSF1R/TAM-directed strategies, or approaches that reduce CAF-mediated immune exclusion [19,20,34,35,36]. For integrin-linked immune resistance, adenosine pathway targeting may be especially relevant, because extracellular adenosine generated through CD39/CD73 can suppress antitumor immunity, and A2A receptor blockade has shown clinical activity in treatment-refractory RCC [37,38]. Our study provides a framework for testing whether ECM-dominant and integrin/adenosine-dominant tumors are associated with different immune resistance mechanisms and show differential responses to these combinations.

This framework may complement existing precision-immunotherapy models in RCC. Prior clinical-translational studies have shown that angiogenesis, T-effector/IFNγ, and myeloid-inflammation signatures can stratify response patterns to immunotherapy and VEGF-targeted therapy in advanced RCC [6]. Combination immunotherapy and VEGF/TKI regimens have improved clinical outcomes, but durable benefit remains heterogeneous, indicating that additional resistance axes are needed for patient stratification [39]. Matrix organization and branch-specific integrin signaling may represent such axes. In particular, ECM-high tumors may be immune-resistant because cytotoxic cells are physically or biochemically constrained by CAF/TGFβ/myeloid/checkpoint networks, whereas RHOA/ROCK-low integrin states may reflect a different tissue organization linked to SRC/ILK and adenosine-associated suppression. Testing these possibilities could help move beyond broad “inflamed” versus “excluded” immune categories toward mechanism-specific support therapies.

Several limitations should be acknowledged. First, most analyses were based on bulk RNA-seq and RPPA, so cell-of-origin and spatial organization cannot be resolved directly. Second, ECM_Stiffness_Core is a transcriptional proxy for matrix remodeling and stiffness-associated ECM biology, rather than a direct measurement of tissue stiffness, collagen alignment, interstitial pressure, or solid stress. Finally, the study did not include an immunotherapy-treated validation cohort, so the translational implications should be treated as a testable direction for future research rather than as claims of clinical response.

Future work should test this model with spatial and functional validation. Spatial transcriptomics, multiplex immunofluorescence, imaging mass cytometry, and second-harmonic collagen imaging could determine whether ECM_Stiffness_Core genes, LOX/fibronectin/collagen programs, CD276, CSF1, TGFB1, adenosine pathway genes, CAFs, macrophages, and exhausted T-cells co-localize within fibrotic intratumoral regions. Direct mechanical assays could clarify whether these regions reflect local stiffness, collagen crosslinking, matrix alignment, or altered pressure, rather than global tumor stiffening. Single-cell and perturbation studies should then separate tumor cell-intrinsic ITGA5/ILK/SRC signaling from CAF-, endothelial-, and myeloid-derived matrix programs. The key translational test will be whether ECM-high CD276/CSF1/TGFB1 tumors and RHOA/ROCK-low integrin/adenosine tumors respond differently to checkpoint blockade plus stromal-, myeloid-, adenosine-, or B7-H3-directed support therapies.

## 5. Conclusions

In summary, our findings support a proposed conceptual model in which ccRCC immune resistance is shaped by matrix architecture and an integrin branch balance rheostat rather than by uniform activation of mechanobiology. Matrix remodeling was associated with a directional suppressive amplifier linked to CAF/TGFβ/CD276/CSF1/VSIR and adenosine-associated immune suppression, whereas integrin-linked mechanosensing separates into a lower-risk PTK2/FAK–RHOA–ROCK branch and a more adverse ITGA5/ILK/SRC-skewed immune-exclusion state. This model reconciles the apparent paradox that matrix remodeling is adverse whereas some canonical focal adhesion components are not, and suggests that matrix and integrin signatures may help distinguish immune-resistant mechanisms for rational combination therapy in ccRCC.

## Data Availability

TCGA-KIRC RNA-sequencing, clinical, survival, and RPPA data are publicly available via the UCSC Xena GDC hub (https://xenabrowser.net) and the Genomic Data Commons (https://portal.gdc.cancer.gov); analysis code and processed intermediate files are available from the corresponding author on reasonable request.

## Supplementary Materials

The following supporting information can be downloaded at: https://www.mdpi.com/article/10.3390/cimb48080789/s1.
