# Supplementary material for: Matrix Architecture and Integrin Branch Balance Distinguish Immune-Regulatory States in Clear Cell Renal Cell Carcinoma
Source: Curr Issues Mol Biol. 2026 Aug 2;48(8):789. doi: 10.3390/cimb48080789 (PMC13511714; doi:10.3390/cimb48080789)
Supplement: Supplementary file 1 [file cimb-48-00789-s001.zip › cimb-4408802-Supplementary Figures.pdf]

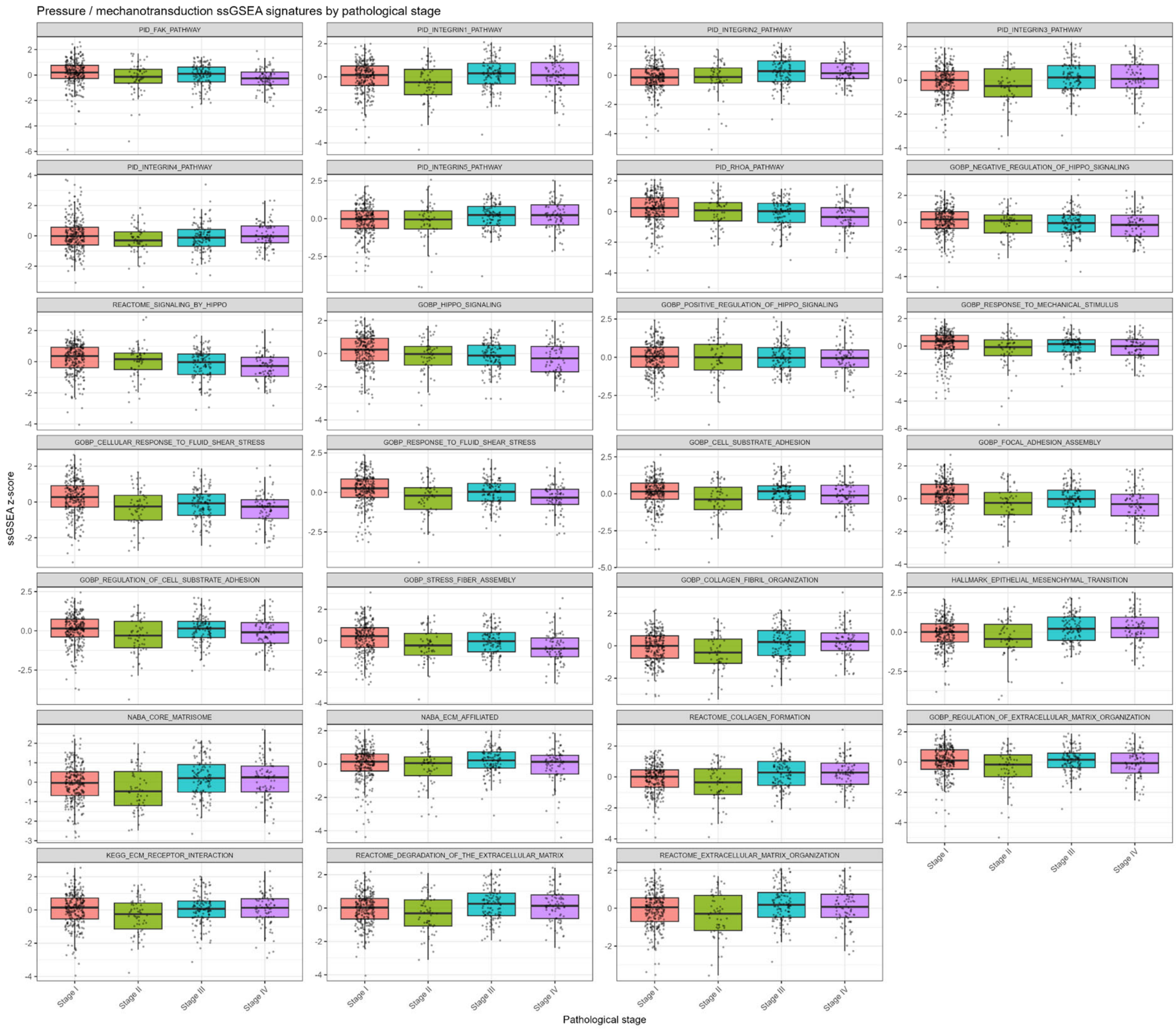

Supplementary Figure S1. Stage-wise distributions of broad pressure, matrix, adhesion, Hippo/YAP, and mechanotransduction ssGSEA signatures in TCGA-KIRC. Boxplots show ssGSEA z-scores for curated pressure- and mechanotransduction-related signatures across pathological stages I–IV. Each panel represents one gene set; boxes indicate median and interquartile range, whiskers indicate the non-outlier range, and points represent individual tumor samples. Signatures include PID FAK, integrin, and RHOA pathways; Hippo/YAP-related pathways; fluid shear stress and mechanical stimulus response signatures; cell–substrate adhesion and focal adhesion assembly signatures; collagen fibril organization; EMT; core matrisome and ECM-affiliated signatures; ECM receptor interaction; degradation of extracellular matrix; and extracellular matrix organization.

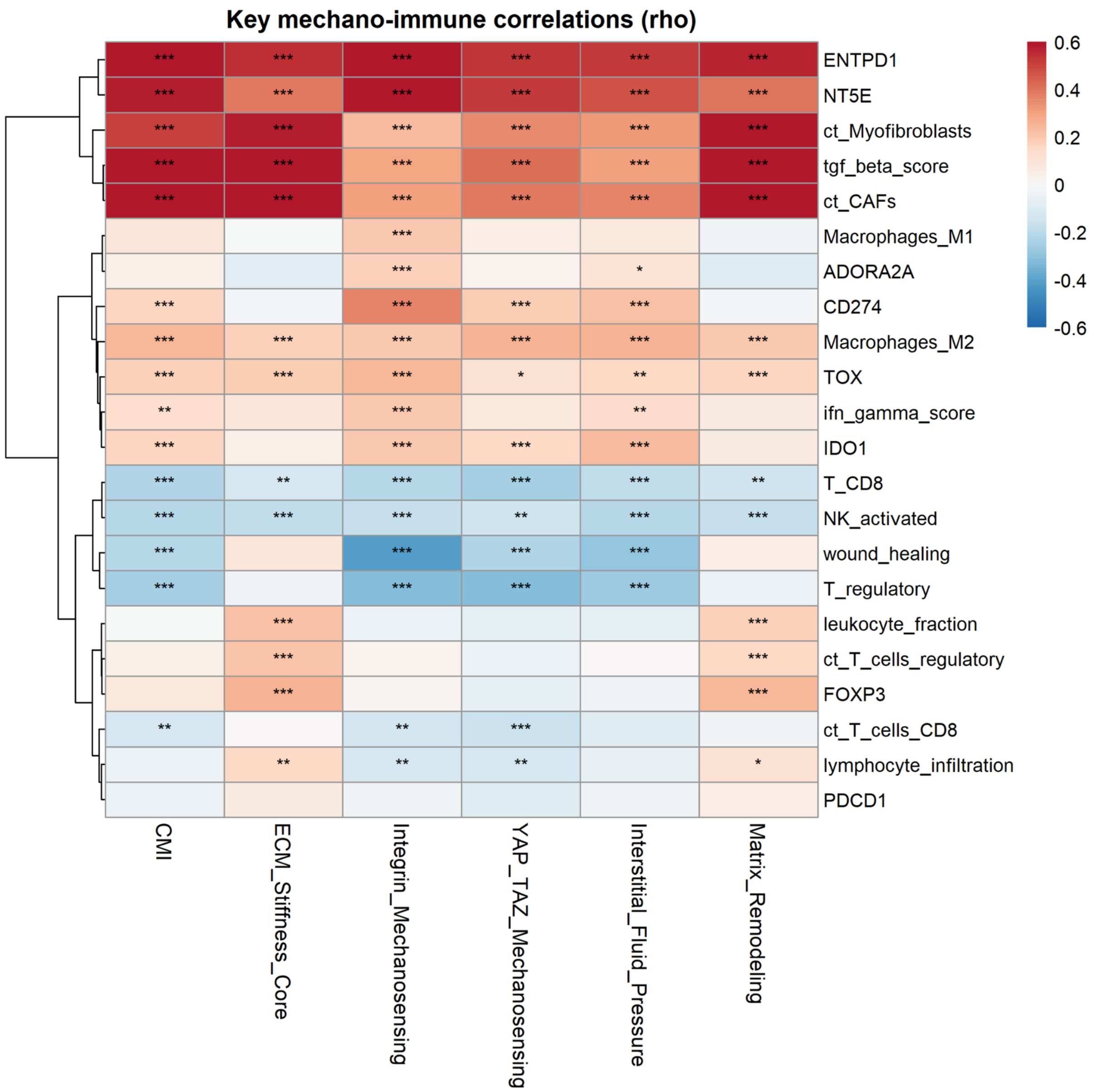

Supplementary Figure S2. Key correlations between mechanobiology signatures and immune-regulatory features. Heatmap showing Spearman correlation coefficients between broad and focused mechanobiology signatures and selected immune-regulatory markers or cell-state scores in TCGA-KIRC. Columns represent mechanobiology signatures, including CMI, ECM_Stiffness_Core, Integrin_Mechanosensing, YAP_TAZ_Mechanosensing, Interstitial_Fluid_Pressure, and Matrix_Remodeling. Rows represent immune features related to adenosine metabolism, TGFβ/stromal activation, myeloid features, checkpoint/exhaustion markers, effector/cytotoxic activity, leukocyte infiltration, and Treg-associated programs. Color indicates Spearman ρ, with red representing positive correlations and blue representing negative correlations. Asterisks indicate statistical significance.

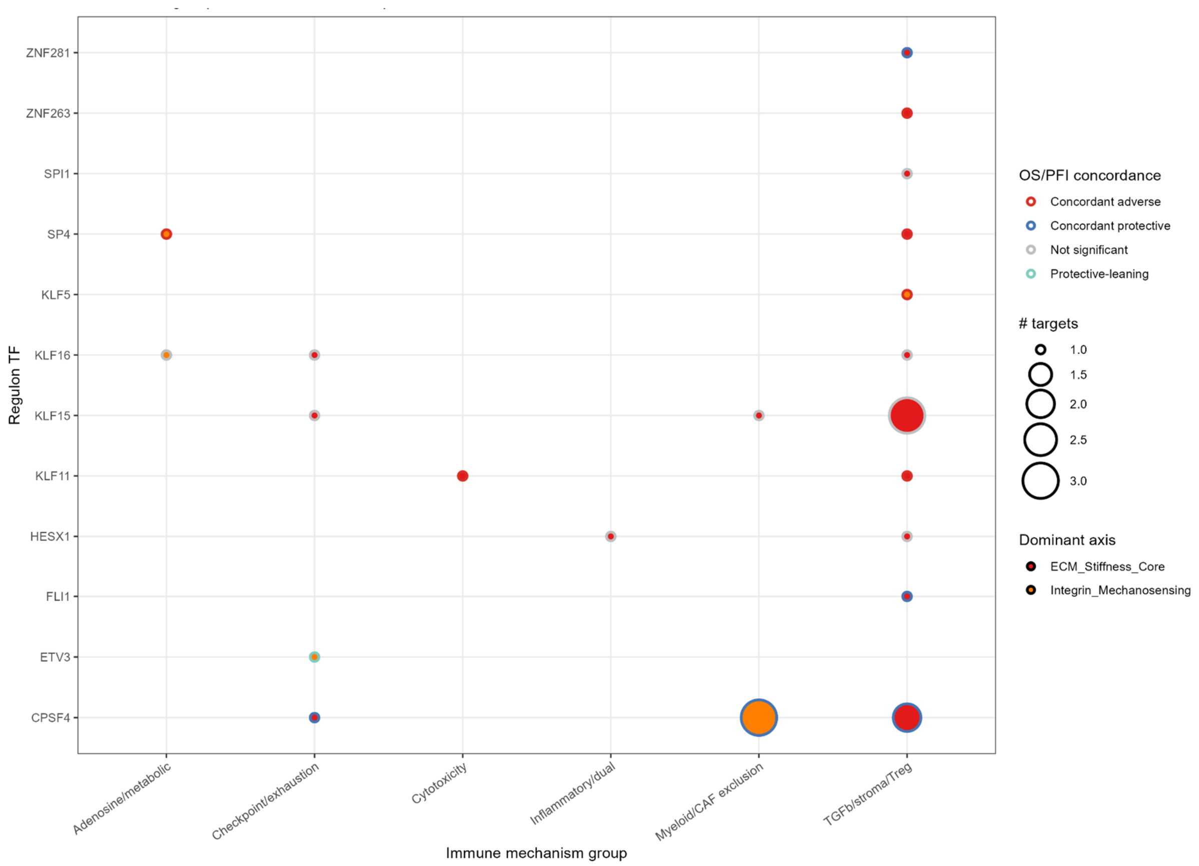

Supplementary Figure S3. TF–immune mechanism coupling within the matrix–integrin regulon network. Dot matrix showing coupling between regulon transcription factors and immune mechanism groups. Rows represent regulon-associated transcription factors and columns represent immune mechanism categories. Dot size indicates the number of immune targets linked to each TF–mechanism pair. Dot fill indicates the dominant mechanobiology axis associated with the TF–immune mechanism relationship, and dot border color indicates OS/PFI concordance category. Mechanism groups include adenosine/metabolic, checkpoint/exhaustion, cytotoxicity, inflammatory/dual, myeloid/CAF exclusion, and TGFβ/stroma/Treg.

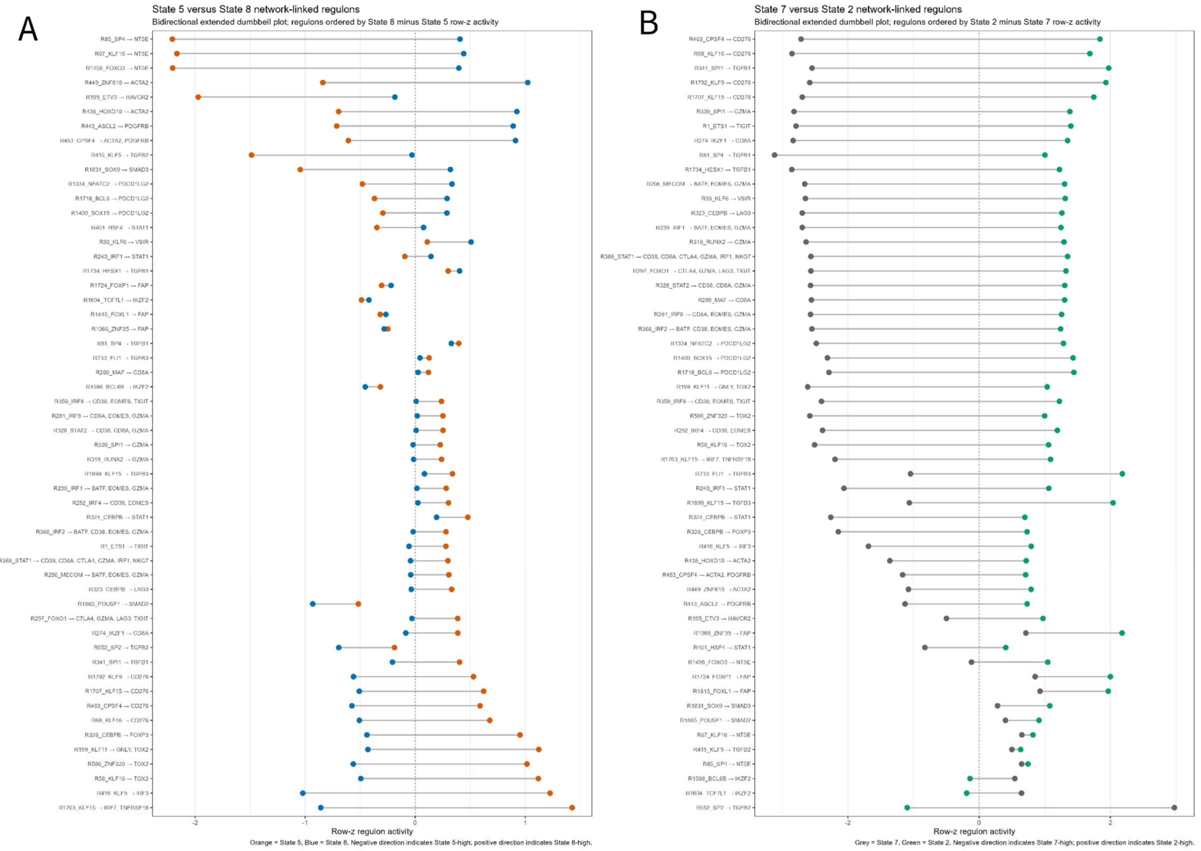

Supplementary Figure S4. State-level activity changes of network-linked regulons. Bidirectional dumbbell plots showing row-z regulon activity differences for network-linked regulons across selected MINER state contrasts. The left panel compares State 5 and State 8 regulons, ordered by State 8 minus State 5 row-z activity. Orange points indicate State 5 activity and blue points indicate State 8 activity. The right panel compares State 7 and State 2 regulons, ordered by State 2 minus State 7 row-z activity. Grey points indicate State 7 activity and green points indicate State 2 activity. Each row represents a regulon–immune target annotation, and connecting lines indicate the difference in activity between the two states. The dashed vertical line marks zero row-z activity.

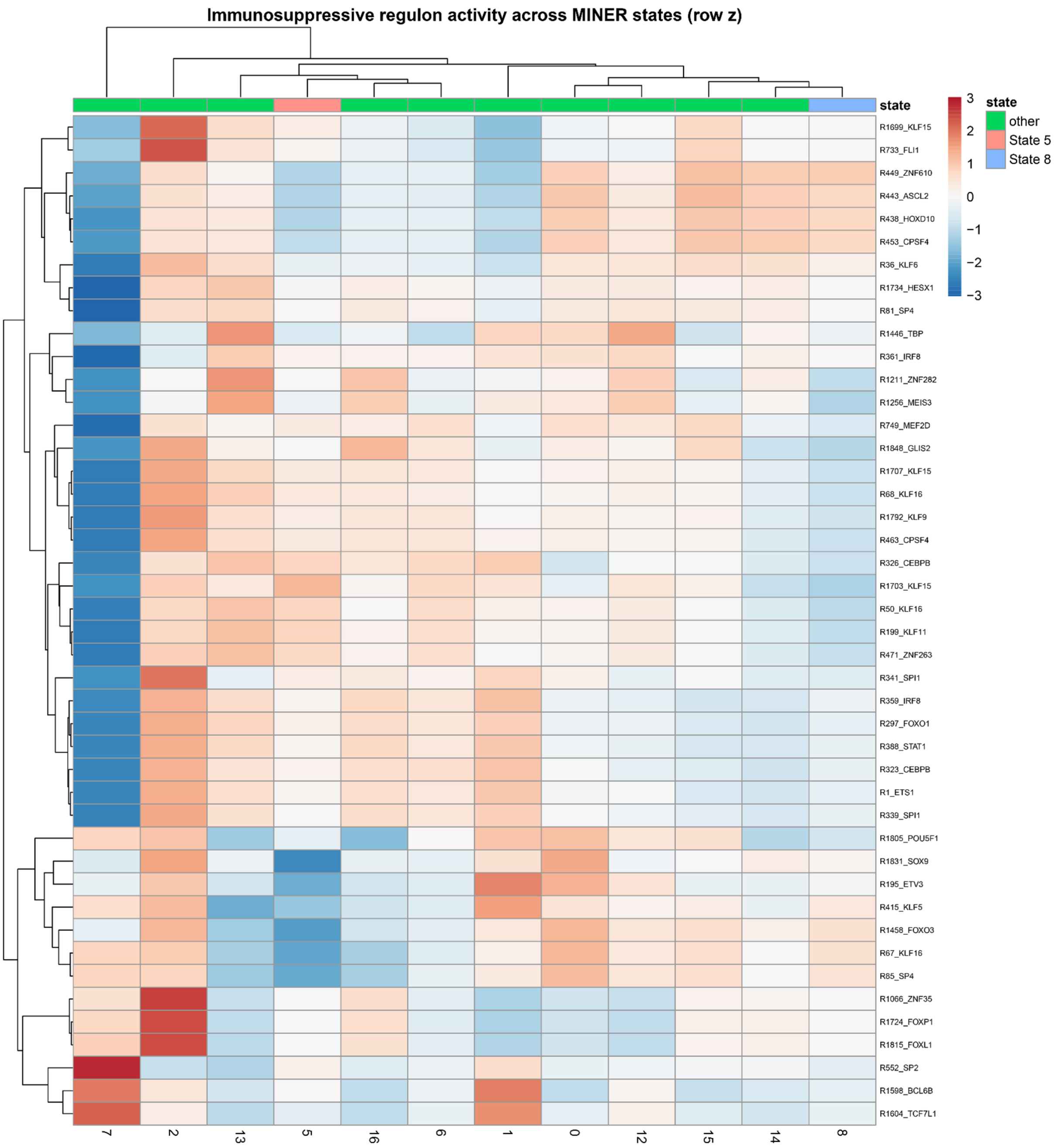

Supplementary Figure S5. Immunosuppressive regulon activity across MINER transcriptional states. Heatmap showing row-standardized activity of immunosuppressive network-linked regulons across MINER states. Rows represent regulons labeled by regulon ID and associated transcription factor, and columns represent MINER transcriptional states. Color indicates row-z-scored regulon activity, with red indicating higher relative activity and blue indicating lower relative activity. Column annotation indicates State 5, State 8, or other MINER states. Row and column dendrograms show hierarchical clustering based on regulon activity patterns.
