# Supplementary material for: Matrix Architecture and Integrin Branch Balance Distinguish Immune-Regulatory States in Clear Cell Renal Cell Carcinoma
Source: Curr Issues Mol Biol. 2026 Aug 2;48(8):789. doi: 10.3390/cimb48080789 (PMC13511714; doi:10.3390/cimb48080789)
Supplement: Supplementary file 1 [file cimb-48-00789-s001.zip › cimb-4408802-Supplementary Tables.pdf]

Supplementary Table S1. Gene Sets and Molecular Signatures Used in This Study

Table S1A. Custom Mechanobiology Signatures (5 signatures)

**ECM_Stiffness_Core**	ECM / Matrix Stiffness	*FN1, COL1A1, COL1A2, COL6A1, COL6A2, COL6A3, LOX, LOXL2, POSTN, TNC*	Custom; fibronectin, fibrillar collagens, lysyl oxidases, matricellular proteins. TEN genes; this full ten-gene set is used in all analyses. The four genes named in the Abstract (FN1, COL1A1, COL6A1, LOX) are representative highest-loading members, not a separate signature. Scored as per-sample mean of gene-wise z-scores. PC1 variance explained: 68.2%.
**Integrin_Mechanosensing**	Integrin / FAK / RhoA	*ITGB1, ITGA5, ITGAV, PTK2, SRC, ILK, RHOA, ROCK1, ROCK2*	Custom; RGD-binding integrins, FAK (PTK2), ILK, RhoA/ROCK effectors. Scored as per-sample mean of gene-wise z-scores. PC1 variance explained: 54.5%. For the branch-balance (rheostat) analysis this axis is decomposed into the two functional sub-branches listed in the next two rows, each scored by the same method.
└ ***ITGA5/ILK/SRC branch***	Integrin sub-branch (upstream)	*ITGA5, ILK, SRC*	Upstream integrin–ILK–SRC arm of Integrin_Mechanosensing. Mean gene-wise z-score. Associated with higher-risk biology and adenosine-linked immune regulation. Note: an earlier version of Figure 4D labeled this branch "ITGA4"; the analyzed gene is ITGA5, a member of the parent signature.
└ ***PTK2/RHOA/ROCK branch***	Integrin sub-branch (downstream)	*PTK2, RHOA, ROCK1, ROCK2*	Downstream FAK–RhoA–ROCK focal-adhesion/actomyosin arm. Mean gene-wise z-score. Shows lower-risk directions, i.e. the opposite prognostic direction to the ITGA5/ILK/SRC branch (the "rheostat").
**YAP_TAZ_Mechanosensing**	YAP/TAZ / Hippo Effectors	*YAP1, WWTR1, CCN2, CCN1, ANKRD1, AMOTL2*	Custom; YAP1/TAZ and canonical transcriptional targets. Mean z-score. PC1 var explained: 47.5%.
**Interstitial_Fluid_Pressure**	Fluid Shear / IFP	*AQP1, VEGFA, PDPN, LYVE1, PROX1*	Custom; aquaporin, VEGF, lymphatic markers. Mean z-score. PC1 var explained: 39.5%.
**Matrix_Remodeling**	Matrix Remodeling / EMT	*MMP2, MMP9, MMP14, TIMP1, TIMP2, TGFB1, ACTA2*	Custom; MMPs, TIMPs, TGFB1, smooth-muscle actin (ACTA2). Mean gene-wise z-score. PC1 variance explained: 54.5%. CAVEAT: this axis contains TGFB1 and ACTA2, so its correlations with the TGF-beta response score and with myofibroblast/CAF features are partly definitional for those two genes. Matrix-versus-CAF independence is therefore established with the non-overlapping ECM_Structural and CAF_Identity sets (Table E), and the key ECM-immune associations were re-tested with TGFB1 and ACTA2 removed (Table G).

All custom signatures, including the two integrin sub-branches, are scored identically: the per-sample mean of gene-wise z-scores of log2(TPM+1) expression. Sensitivity analysis: PC1 of centered and scaled expression, with the PC1 sign oriented to correlate positively with the mean z-score. CMI (Composite Mechanosensing Index) = PC1 across the individual axis scores (64.3% variance explained). For the normal-versus-stage comparison in Figure 1A-C, gene-wise z-scores are computed across the combined adjacent-normal plus tumor sample set so that both tissue types share a common scale; all tumor-only analyses use z-scores computed across tumor samples only.

Table S1B. MSigDB Gene Sets Used for ssGSEA Scoring (33 gene sets)

PID_INTEGRIN1_PATHWAY	Integrin / FAK / RhoA	PID
PID_INTEGRIN2_PATHWAY	Integrin / FAK / RhoA	PID
PID_INTEGRIN3_PATHWAY	Integrin / FAK / RhoA	PID
PID_INTEGRIN4_PATHWAY	Integrin / FAK / RhoA	PID
PID_INTEGRIN5_PATHWAY	Integrin / FAK / RhoA	PID
PID_RHOA_PATHWAY	Integrin / FAK / RhoA	PID
PID_FAK_PATHWAY	Integrin / FAK / RhoA	PID
REACTOME_SIGNALING_BY_HIPPO	Hippo Signaling	Reactome
GOBP_HIPPO_SIGNALING	Hippo Signaling	GO BP
GOBP_POSITIVE_REGULATION_OF_HIPPO_SIGNALING	Hippo Signaling	GO BP
GOBP_NEGATIVE_REGULATION_OF_HIPPO_SIGNALING	Hippo Signaling	GO BP
GOBP_RESPONSE_TO_MECHANICAL_STIMULUS	Mechanical Stimulus / Force	GO BP
GOBP_CELLULAR_RESPONSE_TO_MECHANICAL_STIMULUS	Mechanical Stimulus / Force	GO BP
GOBP_RESPONSE_TO_FLUID_SHEAR_STRESS	Mechanical Stimulus / Force	GO BP
GOBP_CELLULAR_RESPONSE_TO_FLUID_SHEAR_STRESS	Mechanical Stimulus / Force	GO BP
GOBP_CELL_SUBSTRATE_ADHESION	Adhesion / Cytoskeleton	GO BP
GOBP_REGULATION_OF_CELL_SUBSTRATE_ADHESION	Adhesion / Cytoskeleton	GO BP
GOBP_FOCAL_ADHESION_ASSEMBLY	Adhesion / Cytoskeleton	GO BP
GOBP_ACTIN_CYTOSKELETON_ORGANIZATION	Adhesion / Cytoskeleton	GO BP
GOBP_REGULATION_OF_ACTIN_CYTOSKELETON_ORGANIZATION	Adhesion / Cytoskeleton	GO BP
GOBP_STRESS_FIBER_ASSEMBLY	Adhesion / Cytoskeleton	GO BP
REACTOME_EXTRACELLULAR_MATRIX_ORGANIZATION	ECM / Matrix Stiffness	Reactome
REACTOME_DEGRADATION_OF_THE_EXTRACELLULAR_MATRIX	ECM / Matrix Stiffness	Reactome
REACTOME_COLLAGEN_FORMATION	ECM / Matrix Stiffness	Reactome
KEGG_ECM_RECEPTOR_INTERACTION	ECM / Matrix Stiffness	KEGG
NABA_MATRISOME	ECM / Matrix Stiffness	NABA
NABA_CORE_MATRISOME	ECM / Matrix Stiffness	NABA
NABA_ECM_AFFILIATED	ECM / Matrix Stiffness	NABA
GOBP_EXTRACELLULAR_MATRIX_ORGANIZATION	ECM / Matrix Stiffness	GO BP
GOBP_COLLAGEN_FIBRIL_ORGANIZATION	ECM / Matrix Stiffness	GO BP
GOBP_REGULATION_OF_EXTRACELLULAR_MATRIX_ORGANIZATION	ECM / Matrix Stiffness	GO BP
GOBP_REGULATION_OF_COLLAGEN_FIBRIL_ORGANIZATION	ECM / Matrix Stiffness	GO BP
HALLMARK_EPITHELIAL_MESENCHYMAL_TRANSITION	EMT	Hallmark

Retrieved from MSigDB via msigdbr (species: Homo sapiens); names as returned by msigdbr (uppercase). Of the 33 sets listed, 4 were not found under these identifiers in the installed MSigDB release (GOBP_CELLULAR_RESPONSE_TO_MECHANICAL_STIMULUS, GOBP_ACTIN_CYTOSKELETON_ORGANIZATION, GOBP_REGULATION_OF_ACTIN_CYTOSKELETON_ORGANIZATION, GOBP_EXTRACELLULAR_MATRIX_ORGANIZATION), leaving 29; GOBP_REGULATION_OF_COLLAGEN_FIBRIL_ORGANIZATION was then removed for insufficient gene coverage (<9 detected genes), and 27 sets were

finally scored by ssGSEA (GSVA v2.0.7, ssgseaParam: minSize = 9, maxSize = 500, alpha = 0.25, normalize = TRUE). Module scores = mean of constituent signature z-scores. These MSigDB sets were used only for the pathway-level survey (Supplementary Figure 1); the custom axis signatures in Table A were NOT scored by ssGSEA.

Table S1C. Immune-Regulatory Gene Panels (curated)

Immune Checkpoint / Inhibitory	**CD274**	PD-L1
Immune Checkpoint / Inhibitory	**PDCD1**	PD-1; also exhaustion marker
Immune Checkpoint / Inhibitory	**CTLA4**	
Immune Checkpoint / Inhibitory	**TIGIT**	
Immune Checkpoint / Inhibitory	**LAG3**	
Immune Checkpoint / Inhibitory	**HAVCR2**	TIM-3
Immune Checkpoint / Inhibitory	**VSIR**	VISTA
Immune Checkpoint / Inhibitory	**PDCD1LG2**	PD-L2
Immune Checkpoint / Inhibitory	**IDO1**	
Immune Checkpoint / Inhibitory	**CD276**	B7-H3
Immune Checkpoint / Inhibitory	**VTCN1**	B7-H4
Adenosine Pathway	**ENTPD1**	CD39; ATP->AMP
Adenosine Pathway	**NT5E**	CD73; AMP->adenosine
Adenosine Pathway	**ADORA2A**	A2A receptor; effector T cell suppression
Adenosine Pathway	**ADORA2B**	A2B receptor; myeloid/stromal
T Cell Exhaustion	**PDCD1**	PD-1
T Cell Exhaustion	**TOX**	Master exhaustion TF
T Cell Exhaustion	**TOX2**	
T Cell Exhaustion	**NR4A1**	Nur77; early exhaustion/anergy
T Cell Exhaustion	**NR4A2**	Nurr1
T Cell Exhaustion	**PRDM1**	BLIMP-1; terminal exhaustion
T Cell Exhaustion	**BATF**	Exhaustion-associated TF
T Cell Exhaustion	**EOMES**	T-bet paralog
T Cell Exhaustion	**TBX21**	T-bet
Treg-Associated	**FOXP3**	Master Treg TF
Treg-Associated	**IL2RA**	CD25
Treg-Associated	**IKZF2**	Helios
Treg-Associated	**IKZF4**	Eos
Treg-Associated	**TNFRSF18**	GITR
Treg-Associated	**TNFRSF9**	4-1BB
Treg-Associated	**IL10**	Treg effector cytokine
Treg-Associated	**TGFB1**	Treg cytokine; also ECM relevant
Co-stimulatory	**CD80**	
Co-stimulatory	**CD86**	
Co-stimulatory	**CD28**	
Co-stimulatory	**TNFRSF4**	OX40
Co-stimulatory	**CD27**	Inverse exhaustion marker

Co-stimulatory	**ICOS**	Treg/Tfh marker
Cytolytic Activity (CYT)	**GZMA**	CYT score = geometric mean z(GZMA, PRF1)
Cytolytic Activity (CYT)	**PRF1**	
T-cell Inflamed GEP (Ayers 2017)	**CCL5**	
T-cell Inflamed GEP (Ayers 2017)	**CD27**	
T-cell Inflamed GEP (Ayers 2017)	**CD274**	PD-L1
T-cell Inflamed GEP (Ayers 2017)	**CD276**	B7-H3
T-cell Inflamed GEP (Ayers 2017)	**CD8A**	
T-cell Inflamed GEP (Ayers 2017)	**CMKLR1**	
T-cell Inflamed GEP (Ayers 2017)	**CXCL9**	
T-cell Inflamed GEP (Ayers 2017)	**CXCR6**	
T-cell Inflamed GEP (Ayers 2017)	**HLA-DQA1**	
T-cell Inflamed GEP (Ayers 2017)	**HLA-DRB1**	
T-cell Inflamed GEP (Ayers 2017)	**HLA-E**	
T-cell Inflamed GEP (Ayers 2017)	**IDO1**	
T-cell Inflamed GEP (Ayers 2017)	**LAG3**	
T-cell Inflamed GEP (Ayers 2017)	**NKG7**	
T-cell Inflamed GEP (Ayers 2017)	**PDCD1LG2**	PD-L2
T-cell Inflamed GEP (Ayers 2017)	**PSMB10**	
T-cell Inflamed GEP (Ayers 2017)	**STAT1**	
T-cell Inflamed GEP (Ayers 2017)	**TIGIT**	
CD8/FOXP3 Balance	**CD8A**	Effector proxy
CD8/FOXP3 Balance	**FOXP3**	Suppressor proxy

Panels scored from log2(TPM+1) expression or gene-wise z-scores. CYT = mean expression of GZMA and PRF1. GEP = 18-gene T-cell-inflamed gene expression profile (Ayers et al., J Clin Invest 2017). CD8/FOXP3 balance = CD8A minus FOXP3 in log2(TPM+1) space. Genes listed under more than one panel (e.g. PDCD1, CD276, TIGIT, LAG3, IDO1, CD27) are deduplicated before scoring.

Table S1D. Cell-Type Marker Panels for GSVA Immune Deconvolution

**T cells (total)**	*CD3D, CD3E, CD2, CD3G*
**CD8 T cells**	*CD8A, CD8B, GZMK, DUSP2, EOMES*
**Cytotoxic lymphocytes**	*GNLY, GZMB, PRF1, GZMA, NKG7, KLRB1*
**Regulatory T cells**	*FOXP3, IL2RA, CTLA4, TIGIT, IKZF2*
**NK cells**	*XCL1, XCL2, NCR1, KIR2DL1, KIR3DL1, FCGR3A*
**B cells**	*CD19, MS4A1, BANK1, CD79A, IGHM*
**Plasma cells**	*IGHG1, MZB1, SDC1, CD38, JCHAIN*
**Monocytes**	*CD14, CD163, CSF1R, FCGR2A, LYZ*
**Macrophages M1**	*CD68, CD80, IL1B, TNF, CXCL10, NOS2*
**Macrophages M2**	*CD163, MRC1, CD206, ARG1, CCL18, IL10*
**Myeloid DC**	*ITGAX, CD209, HLA-DQA1, CLEC9A, XCR1*
**Neutrophils**	*CSF3R, CXCR2, FCGR3B, S100A8, S100A9*
**Mast cells**	*TPSAB1, CPA3, GATA2, KIT, HDC*
**Fibroblasts**	*VIM, COL1A1, COL3A1, PDGFRA, THY1, DCN*
**CAFs (general)**	*FAP, PDPN, ACTA2, COL1A1, COL1A2, PDGFRB, THY1, FN1, POSTN, S100A4*
**Myofibroblasts**	*ACTA2, MYH11, TAGLN, CNN1, PDGFRB*
**Endothelial cells**	*PECAM1, VWF, CDH5, CLEC14A, ESAM, ENG*
**Pericytes**	*RGS5, PDGFRB, NOTCH3, CSPG4, ABCC9*

Applied to the tumor expression matrix (log2[TPM+1]) via GSVA. Supplemented by published MCP-counter and xCell cell-type scores and by CIBERSORT estimates from the Thorsson et al. PanImmune resource. Note that the CAF and Myofibroblast marker panels deliberately include structural ECM genes (COL1A1, COL1A2, FN1, POSTN, ACTA2); correlations between these deconvolution scores and the matrix signatures are interpreted with that overlap in mind (Table G).

Table S1E. ECM/CAF Disentanglement Gene Sets

**ECM_Structural (clean)**	*COL1A1, COL1A2, COL6A1, COL6A2, COL6A3, FN1, LOX, LOXL2, POSTN, TNC*	Zero overlap with CAF_Identity. Used for mutual partial Spearman vs CAF.
**CAF_Identity (clean)**	*PDGFRA, PDGFRB, FAP, PDPN, THY1, DCN, LUM, COL11A1, MMP11, WISP1, CXCL12, CXCL14*	Zero overlap with ECM_Structural. Fibroblast lineage/activation markers only.
**myCAF (original)**	*ACTA2, MYH11, TAGLN, PDGFRB, FAP, POSTN, LRRC15, HOPX*	Shares ACTA2 with Matrix_Remodeling (Jaccard = 0.071) and POSTN with ECM_Structural (Jaccard = 0.059). Documented as a transparency measure; these overlaps are the reason the clean sets above are used for the decisive tests.
**iCAF (original)**	*IL6, CXCL12, CXCL14, HAS1, CFD, LMNA, IL1B, PDGFRA*	Inflammatory CAF subtype. Shares CXCL12, CXCL14 and PDGFRA with CAF_Identity (Jaccard = 0.176); no overlap with Matrix_Remodeling or ECM_Structural.
**Matrix_Remodeling (orig.)**	*MMP2, MMP9, MMP14, TIMP1, TIMP2, TGFB1, ACTA2*	Same as the custom signature in Table A. Shares ACTA2 with myCAF; shares no genes with ECM_Structural, CAF_Identity or iCAF.

ECM_Structural and CAF_Identity were verified in code to share zero genes (overlap = 0). Both are scored as the mean of gene-wise z-scores. Mutual partial Spearman correlation (ppcor::pcor.test, method = "spearman") was used to determine which program retains an independent association with each immune endpoint after adjustment for the other, complemented by linear-model variance partitioning into ECM-unique, CAF-unique and shared components. Overlap with the original myCAF/iCAF signatures is quantified in Table G.

Table S1F. RPPA Mechanoactivation Ratio Definitions

**FAK_activation**	FAK_pY397 - FAK	Tier 1	Autophosphorylation site; canonical FAK activity
**SRC_activation**	SRC_pY416 - SRC_pY527	Tier 1	Y416 = active; Y527 = inhibitory
**YAP_activity**	YAP - YAP_pS127	Tier 1	Unphosphorylated YAP = nuclear/active
**Myosin_contractility**	MYOSINIIA_pS1943 - MYOSINIIA	Tier 1	Non-muscle myosin IIA contractility
**AKT_activation**	AKT_pS473 - AKT	Tier 1	
**mTOR_activation**	MTOR_pS2448 - MTOR	Tier 1	
**GSK3_inhibition**	GSK3ALPHABETA_pS21S9 - GSK3ALPHABETA	Tier 2	Phospho = inhibited GSK3; downstream of AKT
**EphA2_canonical**	EphA2_pY588 - EphA2	Tier 2	Ligand-dependent / tumor suppressive
**EphA2_noncanonical**	EphA2_pS897 - EphA2	Tier 2	AKT-dependent; pro-migratory
**CMAS (PC1)**	PC1 of all 6 Tier-1 ratios	Composite	PC1 var explained = 31.2%; oriented to correlate positively with FAK_activation; n=260

RPPA data from UCSC Xena: TCPA/MD Anderson Level-4 replicate-base-normalized values (487 antibodies x 478 samples, adjacent-normal plus Stage I-IV tumors; 475 staged tumors). Each antibody is SuperCurve-fitted and median-centered twice (per protein across samples, then per sample across proteins), placing all antibodies on a common dimensionless log2 scale; ratios are therefore computed as simple differences in log2 space (= log2 fold-differences) with no additional per-antibody standardization. Ratio scores were standardized to unit SD before Cox modeling. CMAS = PC1 of the six Tier-1 ratios (n = 260 tumors with complete Tier-1 coverage; PC1 variance explained = 31.2%), oriented to correlate positively with FAK_activation.

Supplementary Table S2. Gene Overlap Between Matrix, Integrin, and CAF Gene Sets

Gene Set A	Gene Set B	Shared Genes	Jaccard
Matrix_Remodeling	ECM_Structural (clean)	*none*	0.000
Matrix_Remodeling	CAF_Identity (clean)	*none*	0.000
Matrix_Remodeling	myCAF (original)	***ACTA2***	**0.071**
Matrix_Remodeling	iCAF (original)	*none*	0.000
ECM_Structural (clean)	CAF_Identity (clean)	*none*	**0.000**
ECM_Structural (clean)	myCAF (original)	***POSTN***	0.059
ECM_Stiffness_Core	Integrin_Mechanosensing	*none*	0.000
ECM_Stiffness_Core	Matrix_Remodeling	*none*	0.000

Key points: (1) ECM_Structural and CAF_Identity share no genes (Jaccard = 0), so the matrix-versus-CAF disentanglement (mutual partial Spearman correlation and variance partitioning) is free of shared-gene circularity. (2) Matrix_Remodeling shares only ACTA2 with myCAF and no genes with CAF_Identity, ECM_Structural or iCAF. (3) Its correlations with the TGF-beta response score and with myofibroblast features

are nonetheless partly definitional through TGFB1 and ACTA2; these associations were therefore re-tested with both genes removed, and the direction and significance of the reported relationships were preserved.

Supplementary Table S3. Per-state Sample Sizes, Survival-evaluable Counts, and Event Counts for MINER Transcriptional States in TCGA-KIRC

State ID	Samples	OS (n)	OS Events (n)	OS Censored (n)	PFI (n)	PFI Event (n)	PFI Censored (n)
5	97	97	54	43	94	50	44
0	92	91	28	63	89	23	66
8	48	48	8	40	47	8	39
14	31	31	5	26	28	6	22
12	30	30	12	18	30	7	23
11	25	25	4	21	25	7	18
6	24	24	8	16	22	10	12
7	22	21	5	16	20	2	18
1	20	20	7	13	20	7	13
20	18	17	4	13	16	5	11
9	17	17	2	15	16	5	11
15	15	14	6	8	13	4	9
17	15	15	3	12	15	4	11
2	14	14	7	7	14	8	6
10	13	13	1	12	13	1	12
13	11	11	5	6	8	3	5
16	10	10	5	5	8	3	5
4	8	8	2	6	8	1	7
21	7	7	3	4	7	2	5
22	7	7	4	3	7	4	3

18	6	6	0	6	6	0	6
3	5	5	0	5	5	0	5
19	3	3	0	3	3	0	3

Tumors were assigned to transcriptional states by MINER3 from the KIRC regulon eigengene matrix; adjacent-normal samples were excluded. For each state, "Tumors" gives the number of assigned tumor samples, "OS" and "PFI evaluable" give the numbers with complete overall-survival and progression-free-interval records, and "OS events" and "PFI events" give the numbers of observed events. These counts are computed by grouping tumors by state and are therefore independent of any reference state used in downstream survival models. The ECM, Integrin, and Matrix columns give the per-state median scores of the ECM_Stiffness_Core, Integrin_Mechanosensing, and Matrix_Remodeling signatures (per-sample mean of gene-wise z-scores). These show that the state contrasts examined in the main text correspond to the extremes of these axes, with States 5 and 8 at opposite ends of Integrin_Mechanosensing and States 7 and 2 at opposite ends of the matrix-associated axes.
